# Genetic profiles of 103,106 individuals in the Taiwan Biobank provide insights into the health and history of Han Chinese

**DOI:** 10.1038/s41525-021-00178-9

**Published:** 2021-02-11

**Authors:** Chun-Yu Wei, Jenn-Hwai Yang, Erh-Chan Yeh, Ming-Fang Tsai, Hsiao-Jung Kao, Chen-Zen Lo, Lung-Pao Chang, Wan-Jia Lin, Feng-Jen Hsieh, Saurabh Belsare, Anand Bhaskar, Ming-Wei Su, Te-Chang Lee, Yi-Ling Lin, Fu-Tong Liu, Chen-Yang Shen, Ling-Hui Li, Chien-Hsiun Chen, Jeffrey D. Wall, Jer-Yuarn Wu, Pui-Yan Kwok

**Affiliations:** 1 Institute of Biomedical Sciences, Academia Sinica, Taipei, Taiwan; 2 Institute for Human Genetics, University of California, San Francisco, CA USA; 3 Department of Genetics, Stanford University, Stanford, CA USA

**Keywords:** Population genetics, Genetics

## Abstract

Personalized medical care focuses on prediction of disease risk and response to medications. To build the risk models, access to both large-scale genomic resources and human genetic studies is required. The Taiwan Biobank (TWB) has generated high-coverage, whole-genome sequencing data from 1492 individuals and genome-wide SNP data from 103,106 individuals of Han Chinese ancestry using custom SNP arrays. Principal components analysis of the genotyping data showed that the full range of Han Chinese genetic variation was found in the cohort. The arrays also include thousands of known functional variants, allowing for simultaneous ascertainment of Mendelian disease-causing mutations and variants that affect drug metabolism. We found that 21.2% of the population are mutation carriers of autosomal recessive diseases, 3.1% have mutations in cancer-predisposing genes, and 87.3% carry variants that affect drug response. We highlight how TWB data provide insight into both population history and disease burden, while showing how widespread genetic testing can be used to improve clinical care.

## Introduction

Over the last two decades, several large, population-based biobanks have been set up to collect blood and other biospecimens together with a standard set of clinical data to power genetic studies of many common diseases^1–5^. The participants are followed up in regular intervals for further biospecimen collection and health examinations, for up to 30 years in some biobanking programs. Several biobanks have completed their cohort collection and released their data for analysis, leading to many new insights into the genetic factors associated with common diseases^4^. A common focus of the early population-based biobanks has been to identify genetic variants associated with disease without considering how the results can be returned to the participants for their own health management. Since many disease-causing mutations are rare and population specific^6–9^, the genetic basis of disease susceptibility varies across populations, which in turn has helped motivate the development of biobanks around the world. As part of the Taiwan Biobank (TWB, established in 2012), a cohort of 200,000 individuals from the general Taiwanese population with no cancer diagnosis at the time of enrollment is being recruited and followed at regular intervals. The majority of Taiwanese are Han Chinese (over 99%) immigrated from different provinces of China and minority of them are Taiwanese aboriginals. Additional facets of the project include an East Asian-focused reference panel for genotype imputation based on high-coverage whole-genome sequencing (WGS) from 1445 early TWB participants, and the development of two custom single nucleotide polymorphism (SNP) genotyping arrays that generate data not only for future genome-wide association studies (GWAS) or polygenic risk score (PRS) development, but also for directly conducting thousands of genetic tests on the cohort.

In this study, we present the WGS data as well as genotyping results from the first 103,106 participants of the TWB. This is the largest publicly available genetic database of individuals with East Asian ancestry. We document the extent to which the population is affected by known risk variants, and show how these results can be used to immediately improve the clinical care of the participants. Further, we highlight the utility of our reference panel for imputation, confirm that our samples provide adequate coverage of genetic diversity across all Han Chinese, and conduct basic population genetic studies of population structure and recent changes in population size in the TWB cohort. Overall, the TWB provides foundational genomic resources that will enable future large-scale genetic studies in individuals closely related to Han Chinese.

## Results

### Overview

The TWB database provides three novel features that increase its utility: (1) high-coverage WGS data from more than 1400 Han Chinese individuals, (2) a custom SNP array that utilizes both previously identified functional variants and the unique linkage disequilibrium structure of Han Chinese, and (3) SNP array data (with linked phenotypic data) from 103,106 TWB participants. We describe the benefits of each of these in greater detail below.

### Han Chinese reference panel

**Fig. 1:**
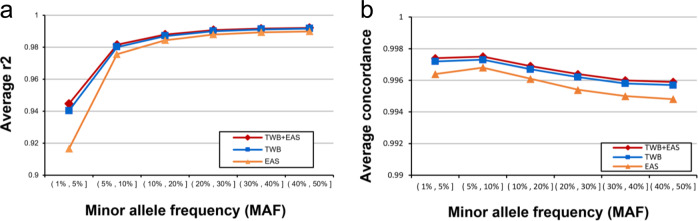
Comparison of imputation performance of the TWBv2 array using three reference panels. The average *r*^2^ values (**a**) and concordance (**b**) are plotted against the minor allele frequency. EAS: reference panel with EAS from 1000 genome; TWB: reference panel with Taiwan biobank NGS data.

To aid in the genotype imputation of East Asian samples in general and TWB samples in particular, we generated high-coverage whole-genome sequence data from 1,445 TWB participants and created a (computationally) phased reference panel (TWB panel) from these data. We then utilized in silico experiments with high-coverage whole-genome sequence data from 137 additional Han Chinese individuals to quantify the imputation accuracy of the TWB panel for all variants with minor allele frequency (MAF) > 0.01. For comparison, we also evaluated the imputation accuracy of the East Asian subgroup of the 1000 Genomes Project (EAS panel, *n* = 504) as well as a combined TWB + EAS reference panel that included the 1949 genomes from the two different groups (The 137 test samples were not included in any of these panels). Figure 1 shows the genotype concordance and the squared correlation coefficient (*r*^2^) between predicted and actual genotypes, as a function of minor allele frequency. We find that the TWB panel provides a modest, but consistent, improvement in imputation accuracy over the EAS panel, and that the TWB + EAS panel provides a very small improvement over the TWB panel. These results are consistent with previous studies showing that imputation accuracy depends on both sample size and genetic similarity between reference panel and genomes to be imputed^3,10–12^. We also find an improvement in imputation accuracy when the reference panel is fixed but the SNP array used varies between the custom TWBv2 array (described below) and the commonly used Illumina GSAv2 array (Supplementary Fig. 1).

### Custom SNP array

The structure of linkage disequilibrium is known to vary across continental populations^13,14^. As a consequence, commercial SNP arrays that were developed for European populations are less effective at detecting true genotype–phenotype associations when applied to non-European groups. In part because of this, we developed two custom genotyping arrays using the ThermoFisher Axiom platform. Since the TWBv1 array was described previously^15^, we focus below on the TWBv2 array (686,463 markers), which was used on >76,000 TWB participants.

The TWBv2 array utilized WGS data from TWB participants to choose SNPs optimized for imputation in Han Chinese samples^16^, contained 114,000 risk variants in 2831 rare disease genes selected from published literature and the ClinVar database, 4100 variants associated with drug metabolism and adverse drug reactions, and 24,865 copy number variation (CNV) probes corresponding to known chromosomal aberrations and CNV regions (Supplementary Tables 1 and 2, Supplemental Text). The array design allowed us to simultaneously assay thousands of actionable functional variants, while also enabling more efficient future GWAS. Overall, 660,606 markers of the TWBv2 array passed quality control, and a comparison of samples with both WGS and array data found an average concordance rate of 99.75% (Supplementary Table 3). Furthermore, we successfully detected 40 out of 41 known CNVs, ranging in size from 108 Kb to 26 Mb (Supplementary Table 4, Supplemental Methods). The only CNV not detected by the TWBv2 array is located at the telomere of Chr1p. It has been split into multiple pieces in the GRCh38/hg38 genome assembly, which leads to CNV call failure.

### Genotyping the TWB cohort

We genotyped 103,106 TWB participants using one or both of our custom SNP arrays, then used the TWB reference panel to impute all biallelic SNPs with MAF > 0.01. TWB recruitment did not target families, but we identified from the genetic data a total of 27,623 relative pairs (3rd degree or closer) involving 34,823 (33.8%) unique individuals (Table 1). These could be divided into 13,238 family groups, including a relatively even distribution of types of relative pairs, suggesting that the TWB participants often invited their close relatives to join the project. This increases the potential utility of the TWB to study the genetic basis of disease susceptibility across all diseases included within the self-reported questionnaire. Our data also include 1171 inferred parent-child pairs, complete with sex and age information, which can be used to verify the accuracy of the TWBv2 array for genetic testing applications.

**Table 1:** Kinship distribution of 34,823 related individuals in the TWB cohort.

Number of members in each group	Number of kindreds with each group size	Number of related pairs				
		MZ twins^a^	Parent-offspring	Full siblings	2nd degree relatives	3rd degree relatives
2	8657	33	1671	2545	2003	2405
3	2686	13	2039	1696	1372	1487
4	1043	11	1518	1037	945	1016
5	415	4	836	657	594	622
6	202	1	492	439	439	440
7	102	0	311	211	284	269
8	54	2	167	121	186	208
9	28	0	102	89	111	148
10	21	0	58	87	117	133
11	12	0	61	34	65	73
12	5	0	31	26	31	33
13	3	1	11	11	15	29
14	4	0	17	18	29	35
15	3	0	16	15	28	32
16	0	0	0	0	0	0
17	2	1	9	7	19	21
18	1	0	6	7	13	10
Total	13238	66	7345	7000	6251	6961

^a^Some of the genetically identical samples could represent duplicates.

**Fig. 2:**
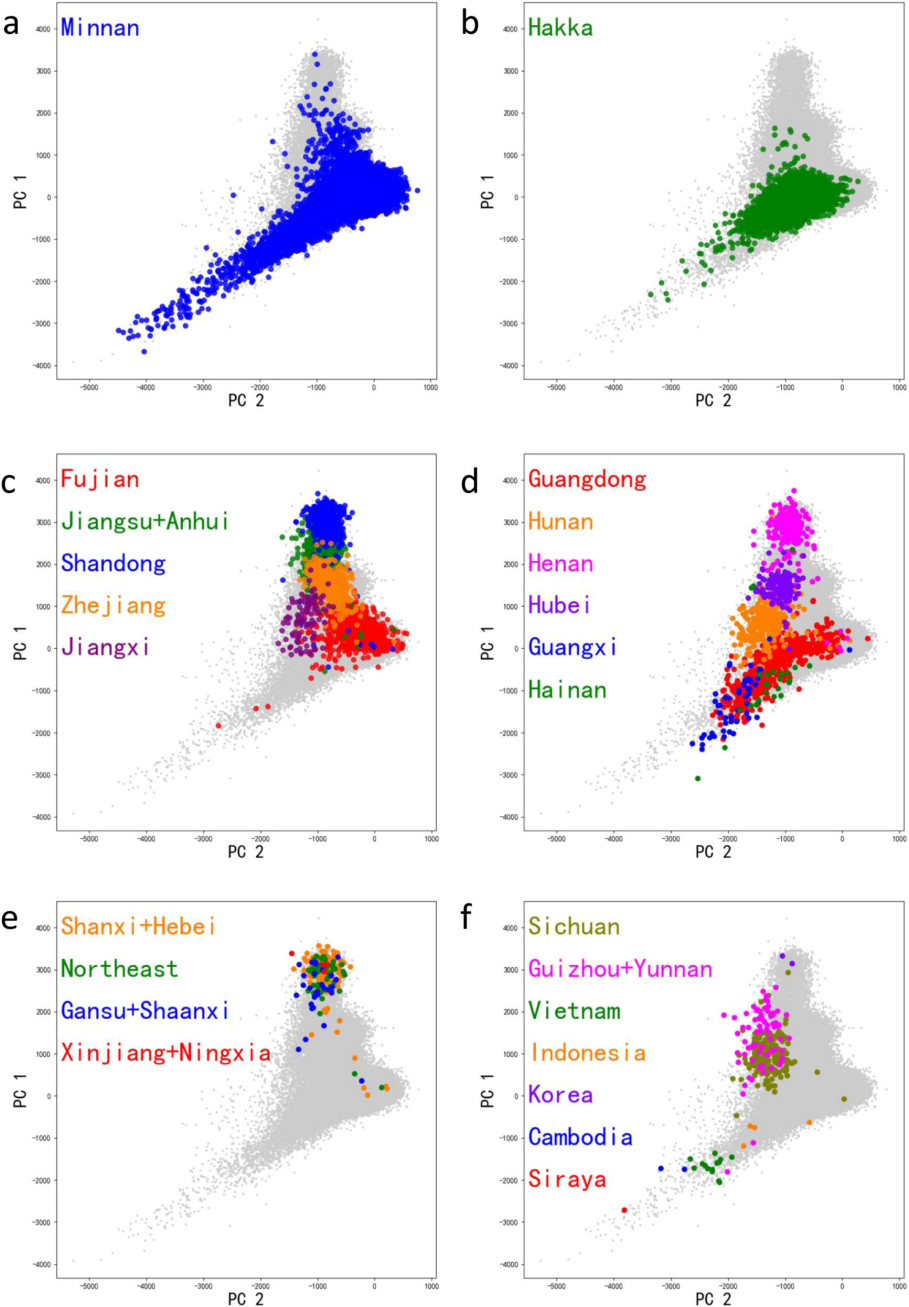
Ancestral diversity of the TWB participants. Clustering of samples from **a** Minnan, **b** Hakka, **c** East China, **d** South Central China, **e** North and Northeast China, and **f** Southwest China + other East Asian groups.

We then used principal components analysis (PCA) to obtain a rough overview of population structure within the TWB cohort (Fig. 2). Our previous work found that over 99% of TWB participants are Han Chinese, including Taiwanese Minnan, Taiwanese Hakka, and people with ancestry from across China^15^. Here, similar to previous studies^17,18^, we found that subjects with both parents from the same province in China clustered together, and that the TWB participants cover the full range of Han Chinese genetic variation. Using the same PCA coordinates, 1000 Genomes Project samples from East Asia (CDX, CHB, CHS, JPT, and KHV) cluster with the TWB samples (Supplementary Fig. 2a), and one self-identified Siraya (an indigenous Taiwanese group) individual from the TWB clusters with known indigenous Taiwanese samples (Supplementary Fig. 2b). Of note, the PCA results based on TWBv1 and TWBv2 arrays are identical.

### Demographic analyses

**Fig. 3:**
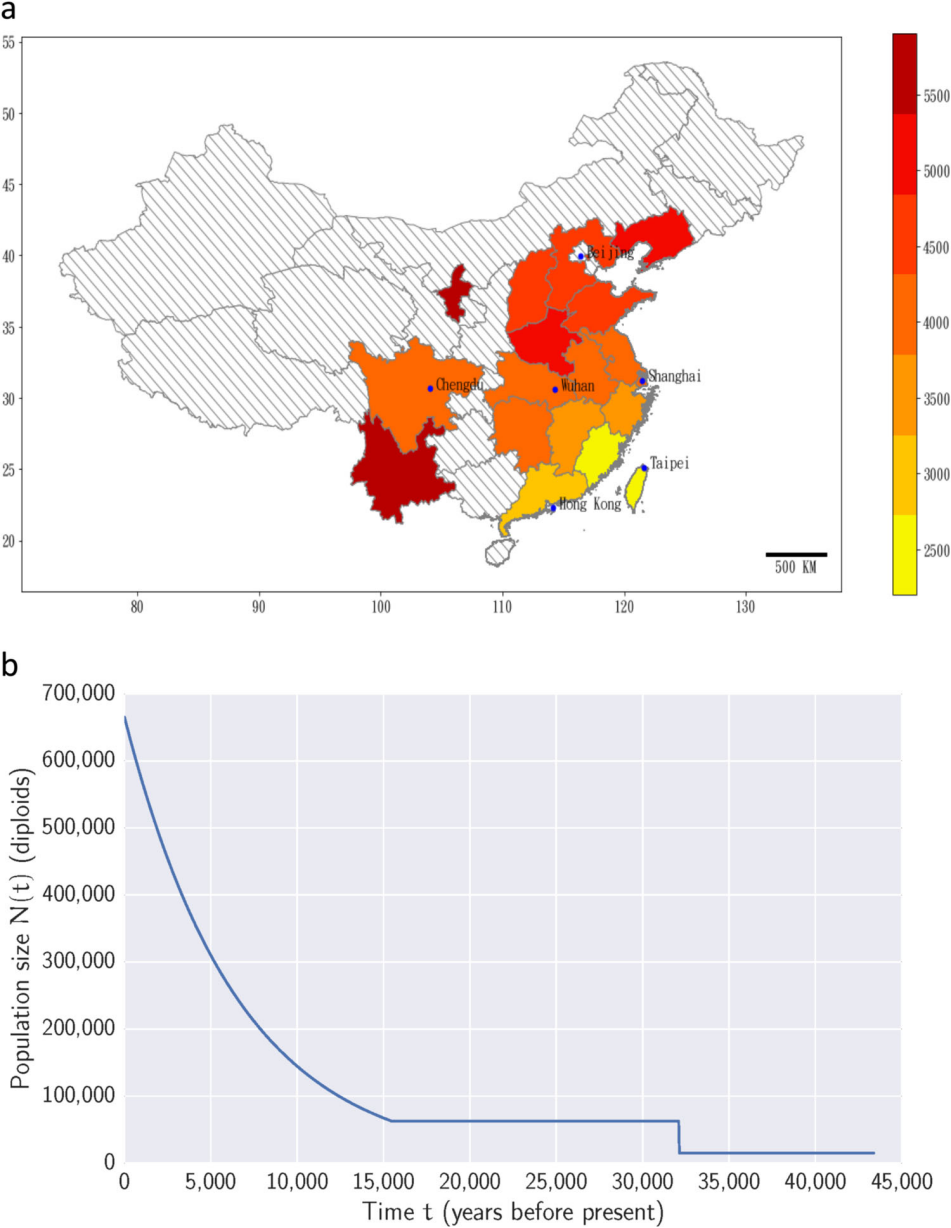
Population substructure and population growth estimates. **a** Number of novel (non-Taiwanese Minnan) variants for each additional sample stratified by province (axes markings represent latitudes and longitudes). Adapted from the Digital Map Database of China, 2020, “Provincial Boundary”, https://doi.org/10.7910/DVN/DBJ3BX, Harvard Dataverse, V1. **b** Estimated past population sizes for Taiwanese Minnan.

The availability of high-coverage WGS data from 1445 TWB participants allowed us to look more closely at population structure and historical changes in population size in our cohort. We focused on self-identified Minnan individuals (who speak a dialect from Southern Fujian province) as representatives of Han Chinese genetic variation found in Taiwan prior to 1945. Then, for other sequenced samples where both parents migrated (post 1945) from the same province in China, we tabulated how many ‘novel variants’ (i.e., SNPs not found in the Minnan) were present (Fig. 3a). We found that individuals with ancestry from Chinese provinces far from Taiwan had more of these novel variants, and thus greater genetic differentiation from Taiwanese Minnan. This trend of isolation-by-distance is highly significant (*r*^2^ = 0.604, *p* = 1.74 × 10^−26^, Fig. S3)

Previous studies have found evidence for exponential (or faster) population growth in European and African American populations using the site frequency spectrum (SFS)^19,20^. We tabulated the SFS for 804 unrelated Minnan genomes, and estimated recent population growth parameters using fastNeutrino^21^. Assuming a mean generation time of 29 years, we estimate that exponential population growth started roughly 15.5 Kya (95% CI 15.2–15.8 Kya) leading to a current effective population size of 665,443 (95% CI 653–678 thousand, Fig. 3b). This growth model is broadly comparable to previously estimated growth parameters in other populations, though the proportion of singletons among our SNPs (46.5%) is substantially smaller than the proportion predicted under the widely used Tennessen et al. model^6^ (57.2%) for European population growth (as implemented by Vernot et al.^22^). This difference likely reflects less extreme very recent population growth in East Asian populations, as well as methodological differences between the two studies (e.g., whole-genome vs. whole-exome data, and taking a well-defined population vs. an aggregate of multiple, closely related populations).

### Massively parallel genetic testing using the TWBv2 array

By design, the TWBv2 array directly genotypes more than 100,000 functional variants, including mutations causing Mendelian diseases, variants associated with complex disease susceptibility, mutations known to affect drug metabolism, and variants across the HLA region. After annotation and sequence validation, we tabulated the genotype frequencies of disease-causing or pathogenic risk variants with MAF > 0.1% in the TWB participants typed on the TWBv2 array (Tables 2–4 and Supplementary Table 5). Surprisingly, we found that 21.2% of the TWB cohort are carriers of at least one Mendelian recessive disorder. While our dataset confirms the previously published carrier rate of many diseases, there are some notable new findings. For example, we observed a higher than expected carrier rate for mutations in genes associated with rare diseases, such as Nagashima-type palmoplantar keratosis (SERPINB7 rs142859678, AF = 0.72%), primary carnitine deficiency (SLC22A5 rs60376624, AF = 0.28%; rs121908893, AF = 0.18%), phenylketonuria (PAH rs76687508, AF = 0.24%), sitosterolemia (ABCG5 rs119480069, AF = 0.33%), and infantile type of Pompe disease (GAA rs28940868, AF = 0.38%). We also observed higher allele frequencies for several pathogenic variants in autosomal dominant diseases than predicted by disease prevalence estimates (Table 3). One example is the Notch3 rs201118034 (R544C) mutation, where 0.88% of the people in our cohort carry the mutation but the autosomal dominant condition it causes, CADASIL, has an estimated prevalence in Taiwan of 3.8 in 10,000, which is 23 times lower than expected^23–25^. In another example, the frequency of PRSS1 rs387906698 (AF = 0.1%) is also higher than the reported disease prevalence of hereditary pancreatitis (0.0003%)^26^.

**Table 2:** Recessive genetic disorders with the highest carrier rates in the TWB cohort.

Recessive genetic disorder	Gene	Carrier rate
G6PD deficiency	G6PD	2.49%
Citrullinemia type II	SLC25A13	1.94%
Wilson disease	ATP7B	1.77%
Pendred syndrome	SLC26A4	1.70%
Krabbe Disease	GALC	1.67%
Nonsyndromic hearing loss and deafness	GJB2	1.59%
Nagashima-type palmoplantar keratosis	SERPINB7	1.43%
Primary carnitine deficiency	SLC22A5	0.90%
Hereditary spastic paraplegia 5	CYP7B1	0.83%
Congenital hypothyroidism	TSHR	0.71%
Sitosterolemia	ABCG5	0.66%
Beta Thalassemia	HBB	0.59%
Total iodide organification defect	TPO	0.56%
Joubert Syndrome	CEP290	0.51%
Usher syndrome	USH2A	0.50%
Phenylketonuria	PAH	0.48%
Mucolipidosis type III	GNPTAB	0.44%
Waardenburg syndrome	EDNRB	0.40%
Congenital Disorder of Glycosylation 1a	PMM2	0.40%
Glutaric aciduria 1	GCDH	0.38%

**Table 3:** Autosomal dominant disorders with the highest allele frequencies in the cohort.

Dominant genetic disorder	Gene	Allele freq.
DFNA2 nonsyndromic hearing loss	KCNQ4	1.24%
Hereditary pancreatitis	SPINK1, PRSS1	1.05%
Familial hypercholesterolemia	APOB, LDLR	0.89%
Cerebral autosomal dominant arteriopathy with subcortical infarcts and leukoencephalopathy type 1 (CADASIL)	NOTCH3	0.88%
Familial hypertrophic cardiomyopathy	TCAP	0.45%
Spastic paraplegia 4	SPAST	0.29%
Maturity-onset diabetes of the young type 3	HNF1A	0.17%

**Table 4:** Cancer susceptibility conditions with the highest fraction of affected individuals in the cohort.

Cancer susceptibility syndromes	Gene	Allele freq.
Hereditary cancer-predisposing syndrome	CHEK2 + RAD51	0.58%
Juvenile polyposis syndrome	SMAD4	0.44%
Adenomatous polyposis coli	APC	0.42%
Breast-ovarian cancer, familial 1	BRCA1	0.41%
Li-Fraumeni syndrome 1	TP53	0.40%
Lynch syndrome	MSH6	0.40%
MYH-associated polyposis	MUTYH	0.40%
Neuroblastoma	KIF1B	0.40%
PTEN hamartoma tumor syndrome	PTEN	0.40%
Prostate cancer	EHBP1	0.40%
Breast-ovarian cancer, familial 2	BRCA2	0.29%

The TWBv2 array includes deleterious germline variants in several cancer-predisposition genes. Although TWB excluded cancer diagnosed subject at the first interview, we still identified 16 pathogenic variants in 13 genes associated with cancer risk that have an allele frequency of >0.1% in our population, including 6 in genes classified as reportable ACMG secondary findings (SF v2.0). For example, 3 pathogenic variants encoding truncated non-functional BRCA1 and BRCA2 proteins have allele frequency >0.1% in our population. Overall, 3.1% of TWB participants carry at least one previously identified cancer-predisposition mutation, and these putative carriers are likely at increased risk for developing cancer in their lifetime (Table 4 and Supplementary Table 5).

We also assessed the allele frequencies of key pharmacogenomic (PG) variants that are known to affect drug metabolism and drug responsiveness in our dataset. 87.3% of all individuals have at least one variant that could affect medication choice or dosage (Table 5 and Supplementary Table 6). As with previous studies^27,28^, we find substantial variation between allele frequencies estimated from the TWB cohort and allele frequencies previously estimated in other populations. For example, the reduced function allele UGT1A1*28 is common in Caucasians^29,30^, whereas the UGT1A1*27 is common in East Asians and Han Chinese in Taiwan.

**Table 5:** Frequency distribution of pharmacogenetic phenotypes predicted by genotypes of TWB cohort.

Gene	Drug	Rx^a^/year	EM	IM	PM	ADR^b^ carrier rate
CYP2B6	Efavirenz	1,662,525	66.0%	30.5%	3.6%	
CYP2C19	Clopidogrel	63,664,076	39.8%	56.4%	3.8%	
CYP2C9	Celecoxib	65,058,810	93.6%	6.3%	0.1%	
CYP3A5	Tacrolimus	10,272,406	8.1%	40.6%	51.2%	
IL28	Peginterferon	40,941	88.6%	11.1%	0.3%	
NAT2	Isoniazid	7,885,251	28.8%	59.2%	12.0%	
SLCO1B1	Simvastatin	50,695,934	78.9%	19.9%	1.3%	
TPMT	Azathioprine	7,435,217	97.0%	2.9%	0.02%	
UGT1A1	Atazanavir	719,793	53.2%	39.8%	7.0%	
VKORC1	Warfarin	16,121,944	1.1%	19.2%	79.7%	
HLA-A*3101	Carbamazepine	17,078,849				2.0%
HLA-B*1502	Carbamazepine	17,078,849				4.1%
HLA-B*5701	Abacavir	3,049,217				0.2%
HLA-B*5801	Allopurinol	23,888,472				10.5%
MT-RNR1	Amikacin	321,561				4.7%

^a^Rx = prescriptions.

^b^*ADR* = adverse drug reactions.

### Imputation of ABO blood groups and HLA types

The TWBv2 array contains variants that can be used to accurately impute ABO blood groups and HLA types. We estimate that these can be imputed from array data with 99.9% and >97.4% accuracy, respectively, based on Mendelian consistency of the data of parent–child pairs in the cohort (Supplementary Table 7). Furthermore, the cross-validation experiment showed that the accuracies of estimated HLA alleles were better than 91.4% across all loci (Supplementary Table 8).

Using the combination of rs8176719, rs8176746, and rs8176747, we determined that the distribution of genetically determined A, B, O, and AB carriers were 26.5%, 24.4%, 43%, and 6% in TWB cohort, consistent with previous ABO blood-antigen typing results^31^. Since the ABO blood groups are suspected to be associated with various health conditions^32,33^, we analyzed the association between predicted ABO blood groups and self-reported clinical phenotypes in the TWB cohort. We found that blood type O was less likely to be associated with epilepsy, consistent with the findings of a previous study^34^, and that TWB participants with blood type AB had a significantly higher incidence rate of epilepsy compared to type O participants (OR = 1.84, 95% CI ~1.2–2.8).

Several HLA alleles are associated with autoimmune diseases and adverse drug reactions. We found that 5.3% Taiwanese were carriers of HLA-B*27:04, a risk factor for ankylosing spondylitis, and 4.1% of our population had HLA-B*15:02, known to be associated with carbamazepine-induced Stevens–Johnson syndrome (Table 5 and Supplementary Table 6)^35,36^. In addition, we found significant regional variation in some HLA allele frequencies, consistent with previous results using hybridization or the sequencing-based typing method (The Allele Frequency Net Database, see Web Resources). For example, HLA-A*02:06 and HLA-B*31:01 are found predominantly in individuals from Northern China, while HLA-A*02:07 and HLA*B33:03 are common in those from Southern China (Supplementary Fig. 4).

## Discussion

Recently, there has been an increased appreciation for the fact that the public health benefits of genetic studies are greatest in the populations that are directly studied, and that equitable “personalized medicine” will require the development of large-scale genomic resources in a wide range of ancestry groups^35,36^. The Taiwan Biobank was created in part to catalyze future medical genetics studies in Taiwan, and the sample size of individuals with dense SNP array data in the TWB (n = 103,106) is several times larger than from comparable Biobanks in Japan and China^1,3^. In addition, our generation of a large reference panel and development of a custom SNP array makes the resulting TWB genotype data much more valuable than comparable studies that rely on existing European-biased SNP arrays and reference panels for genotyping and imputation^4^. In particular, the Taiwan Biobank array includes thousands of Mendelian disease mutations and known pathogenic variants. So, we can cheaply and efficiently conduct thousands of genetic tests on the participants while simultaneously collecting genetic profiles that can be used for PRS calculations for common diseases and future GWAS.

Our analyses highlight the potential utility of including commercial SNP genotyping (using a custom array) into the standard practices of clinical care. We demonstrate that ABO blood types and HLA types can be accurately inferred from an inexpensive commercial SNP array, which may help their inclusion in future Phewas studies. Surprisingly, we found that 21.2% of the population are carriers of known gene mutations responsible for recessive genetic diseases, 4.7% have known gene mutations causing autosomal dominant diseases, and 3.1% carry known gene variants causing cancer susceptibility. Further, 87.3% of the population carry variants that alter their ability to metabolize commonly prescribed drugs or mark them for susceptibility for severe adverse drug reactions (ADRs). All of this information is of obvious utility for both clinicians and patients. For example, with imputed HLA genotypes available in the patients’ medical record, the physician can prescribe medications to patients without the HLA genotypes responsible for specific drug-induced ADRs with confidence and use alternative medications for patients with the HLA genotypes that put them at risk for ADRs^37–39^.

Interestingly, the population allele frequencies of several pathogenic variants are higher than those predicted by disease prevalence, probably due to incomplete penetrance or previously undiagnosed cases with milder clinical symptoms in these autosomal dominant diseases. For example, patients carrying the CADASIL founder mutation, NOTCH3 R544C, display a much broader clinical spectrum than that of classical CADASIL, which may explain the difference between genetic and clinical diagnosis^40,41^. Interestingly, some older individuals with the NOTCH3 rs201118034 (R544C) mutation have MRI (magnetic resonance imaging) evidence of multifocal brain lesions without clinical symptoms^25^.

Although the original TWB study design does not a provision to return results to the participants, discussions have been initiated to return clinically relevant results, such as cancer risk, to the participants who opt to receive such information for clinical management.

Genetic profiling using SNP arrays have several limitations, including (1) genotype calls for extremely rare variants (MAF < 0.1%) are unreliable, (2) only known variants will be typed so de novo germline mutations and somatic mutations will be missed, and (3) some important variants in duplicated regions in gene families cannot be typed due to lack of probe design options. These platform-specific limitations cannot be overcome and studies of some important variants have to be done by other means. However, while genotyping does not capture all possible risk variants, our results show that the majority of the variants of appreciable frequency can be tested at relatively low cost (~USD 40 from blood to data).

Unlike genome-wide association studies that focus on identifying risk variants for gene discovery and downstream therapeutic development, personalized or precision medicine aims to aggregate all risk factors to predict disease risk for an individual. In this study, we generated a large reference panel that greatly improved the imputation accuracy of SNP genotyping data and designed a custom SNP array optimized for genetic studies in the Han Chinese population, the largest ethnic group that, at 1.5 billion, accounts for 19% of the world’s population. While it is custom-built for the TWB, the TWBv2 array is available to all commercially without any restrictions. Furthermore, we obtained genetic testing results for thousands of known risk variants and simultaneously collected genetic profiles in the TWB participants for PRS calculations for common diseases and future GWAS. As a test that needs to be done only once in a person’s lifetime, it has great clinical value. Overall, our study shows that combining comprehensive genetic testing and returning of results in a population setting can serve as a model for precision health management.

## Methods

### Participant samples

Demographic and health-related survey data for 103,106 individuals, together with WGS data (1492 individuals), genotyping data (27,737 typed on the TWBv1 custom array and 75,369 on the TWBv2 array, with 1463 typed on both), and high-resolution allele typing of 6 HLA alleles (1101 individuals) were obtained from the Taiwan Biobank with the approval from the respective ethical committees of the Academia Sinica and the Taiwan Biobank. In addition, TWBv2 genotype data and high-resolution HLA typing data from 502 individuals and WGS data from 26 individuals were obtained from the Collaborative Study to Establish a Cell Bank and a Genetic Database on Non-Aboriginal Taiwanese^42^. WGS data from 64 individuals were obtained from the Pan-Asian Population Genomics Initiative and the Taiwan Han Chinese Sequence Database. These studies were approved by the ethical committee of Academia Sinica. All data from human participants were obtained from databases where data sharing was part of the consent, so the waiver of consent was granted by the Academia Sinica IRB.

### TWB array design

The TWBv1 SNP array was designed in 2011 for genome-wide association studies and the markers were selected from several sources, including the SNPs on the Axiom Genome-Wide CHB 1 Array plate (Affymetrix, Inc., Santa Clara, CA, USA), with a MAF ≥ 5% based on genotyping results of 1950 Taiwanese Han Chinese samples, exonic SNPs with MAF > 10% based on genotyping results of 600 Taiwanese Han Chinese samples, ancestry informative SNPs^43^, SNPs associated with cancer risk^44^, and SNPs on the Affymetrix DMET pharmacogenetic array. The array consists of a set of ∼650,000 SNPs that was designed to provide maximal coverage (*R*^2^ > 0.8) of the human genome.

The TWBv2 SNP array (Thermo Fisher Scientific, Inc., Santa Clara, CA, USA) was designed in 2017 for both GWAS and testing of known risk alleles. Accordingly, TWBv2 has 106,614 coding sequence variants (vs 9545 in TWBv1) and 92,804 protein-altering variants (vs 5972 in TWBv1). There are 104,463 overlapping markers on the two arrays, of which 98,034 passed QC. Overall, the TWBv2 array contains ~415,000 markers for GWAS and imputation. The GWAS markers were selected from the whole genome sequencing data of 946 TWB participants to optimize for coverage of the Han Chinese in Taiwan. Around 57,000 markers intensively covering 179 known disease-relevant CNV regions were also included in the array (Supplementary Table 2) and all markers on the array were used for whole-genome copy number detection. Among ~214,000 markers associated with known diseases, ~114,000 risk variants designated as pathogenic, likely pathogenic, and high-risk variants were selected from several sources, including ACMG, ClinVar, GWAS Catalog, HGMD, locus-specific databases, and the literature. The rare genetic disease genes and variants included in the array are listed in Supplementary Table 9. The drug metabolism gene variants were selected from the literature and on-line databases (CPIC, PharmVar, and FDA). The full list of variants on the TWBv2 array can be found at https://www.twbiobank.org.tw/new_web/exp_doc/TWBv2.0_SNPs%E4%BD%8D%E9%BB%9E%E7%9B%B8%E9%97%9C%E8%B3%87%E8%A8%8A.zip.

### Imputation

The imputation of the GWAS data from the 103,106 individuals was carried out by a three-step process. The first step is to establish a population-specific reference haplotype panel from 1445 genome sequences of Taiwan Biobank, which were aligned to GRCh38 using alt-aware pipeline of bwa-kit and jointly called using GATK^45,46^. A total of 9,387,945 biallelic variant sites with MAF > 1% were selected for computational phasing, performed using SHAPEIT2. Additional haplotypes derived from the EAS sample of the 1000 Genomes Project were also incorporated to enrich the diversity of the panel. After removing the non-monomorphic sites and using the same estimation process, the EAS reference haplotypes consisted of 30,498,845 mapping sites, of which 8,761,215 have MAF > 1%. There were 8,291,319 variants shared between the Taiwan and EAS reference panels.

The second step is to impute the un-genotyped SNPs/variants based on the reference panels. Pre-phasing and imputation were carried out using SHAPEIT2 and IMPUTE2^47,48^. We “Hard-called” genotypes, calling genotypes if the posterior likelihood was >0.9. If not, the genotype was treated as missing data. The expected dosages were calculated directly from the posterior genotype likelihoods from IMPUTE2.

The third step is to validate the imputed results by comparing between whole-genome sequences and imputed genotypes from 137 independent samples (47 from the TWB cohort, 26 from the Collaborative Study to Establish a Cell Bank and a Genetic Database on Non-Aboriginal Taiwanese project, and 64 from the Pan-Asian Population Genomics Initiative and the Taiwan Han Chinese Sequence Database). The concordance between sequence data and hard imputed genotype calls at each variant was used to validate the imputed result. The squared Pearson correlation (*r*^2^) between sequence and dosages was also calculated.

Finally, we converted the TWBv1 array coordinates from GRCh37 to GRCh38 and performed imputation using the TWB reference panel. An allelic association test of imputed genotypes (based on a χ^2^ test) was used to find the frequency differences between 27,737 TWBv1 samples and 75,369 TWBv2 samples. After filtering out the variants with MAF < 5% and call-rate <90%, 4,596,726 of 5,177,055 (88.8%) were not significant (*p*-value >10^−4^).

For the comparison across genotype arrays (Supplementary Fig. 1), we used the 1000 Genomes Project high-coverage data as a reference panel and constructed in silico SNP array data (both TWBv2 and GSA2) using whole-genome sequence data from the Japanese samples in the GenomeAsia 100 K Project callset^27^. We then compared imputation accuracy stratified by MAF using *r*^2^ as described above.

### Principal component analysis

Principal component analysis (PCA) was performed using a two-stage approach. The first stage was the training stage, which estimated the principal components (linear combinations of allele count of SNPs) using a set of 58,393 autosomal SNPs from 25,000 subjects. To maximize the diversity of the training set, all subjects with at least one parent born in mainland China were selected (*N* = 19,110). The remaining 5890 subjects were randomly sampled from the TWB participants who had both parents born in Taiwan. The SNPs used for PCA were randomly selected from the pool of autosomal SNPs on TWB 2.0 array with the following criteria: minor allele frequency >5%, low inter-marker linkage disequilibrium (*r*^2^ < 0.3), call-rate larger than 99%, and Hardy–Weinberg equilibrium (*p* > 10^−4^). The second stage was to calculate values of principal components for all 96,715 subjects (including the training set of 25,000 subjects) for which survey data on familial origins were available.

### Novel allele analysis

This analysis focused only on those individuals with high-coverage WGS data. We started with the 825 individuals who self-identified as being Taiwanese Minnan and removed one individual from each 1st-degree relative pair as well as obvious genetic outliers. This left us with a panel of 804 Minnanese. 125 out of the remaining WGS samples self-identified as having both parents from the same province in mainland China. For each sample, we counted up the number of SNP alleles present in the sample but not present in the 804 Minnanese. (Note that private homozygous variants were counted twice.) These counts were then averaged across province-of-origin for Fig. 3.

To test for a correlation between number of novel variants and distance from Taiwan, we calculated the distance (in km) between each provincial capital and Taipei and constructed a scatterplot of novel variants vs. distance for the 125 mainland WGS samples. We then calculated the Pearson’s correlation coefficient and assessed its significance using a *t*-test with 123 degrees of freedom.

### Population growth

We considered 804 unrelated Minnan individuals (as described above) and considered all autosomal SNP variation in ‘callable’ regions of the genome using the GIAB mask file (see Web Resources). We tabulated the folded SFS of numbers of sites with various minor allele counts for those variants with high-confidence (GQ ≥ 40) genotype calls in all 804 individuals. We then explored four families of population size change models, containing:

1. A single epoch of constant size [c]
2. A single epoch of exponential growth/decline [e]
3. Two epochs of constant size [cc]
4. One epoch of constant size followed by one epoch of exponential growth/decline [ec].

We then used fastNeutrino^21^ to estimate best-fit parameters for each model family, and then compared the model families to each other using the Bayesian information criterion (BIC). The model with the lowest BIC was the 4th one [ec]. To convert the parameters of this best-fit model into years and effective population size, we assumed a mean generation time of 29 years, a mutation rate of 1.25 × 10^−8^ per site per generation, and an autosomal diversity estimate of π = 6.977 × 10^−4^ per site. Finally, 95% confidence intervals for model parameters were obtained as previously described^21^.

### HLA type prediction

We imputed the classical HLA loci (*HLA*-*A*, *HLA*-*B*, *HLA*-*C*, *HLA*-*DRB1*, *HLA*-*DQB1*, and *HLA*-*DPB1*) at two-field resolution using the Hibag R package^49^ with a 765 sample training set. A 500-kb flanking region along with each HLA locus (including 2446–3496 SNPs) was used for subsequent imputation. The prediction model was evaluated by independent validation samples for *HLA-A*, -*B*, -*C*, -*DPB1*, -*DQB1*, and *-DRB1* genes by cross-validation. The accuracy of imputed HLA types was also estimated by consistency of HLA types of parent–child pairs in the cohort. The frequencies of all predicted HLA loci among 103,106 Han Chinese were calculated. For 75,369 TWB participants genotyped using the TWBv2 array, the input for Hibag prediction was genotype data; we used the imputed genotype data as input for those samples genotyped using the TPMv1 array.

### ABO blood type imputation

We inferred ABO blood types (AA, AO, BB, BO, AB, and O) from three SNPs (rs8176719, rs8176746, and rs8176747) located in the *ABO* gene as described previously^50^. The frequencies of the imputed ABO blood types of TWB participants were compared with those derived from antigen-typed ABO blood types as reported previously^31^. The accuracy of imputed ABO blood types was also estimated by consistency of blood types of parent-child pairs in the cohort.

### Reporting summary

Further information on research design is available in the Nature Research Reporting Summary linked to this article.

## Supplementary information

Supplementary Information

Reporting Summary

## Data Availability

The TWB genetic and phenotype datasets, together with the WGS data from 1445 TWB participants, are available through the TWB (https://www.twbiobank.org.tw/new_web_en/about-export.php). The data that support the findings of this study from “The Pan-Asian Population Genomics Initiative and the Taiwan Han Chinese Sequence Database” and the “Collaborative Study to Establish a Cell Bank and a Genetic Database on Non-Aboriginal Taiwanese” are available through the Taiwan National Center for Genomic Medicine (NCGM, http://ncgm.sinica.edu.tw/ncgm_02/contact_e.html) upon request. The GenomeAsia 100K Project callset is available on request (https://genomeasia100k.org/collaborate). The high-coverage NGS data of 1000 Genomes Project used in this study is available in http://ftp.1000genomes.ebi.ac.uk/vol1/ftp/data_collections/1000G_2504_high_coverage/working/20190425_NYGC_GATK/.

## Code availability

No custom computer code was used in this study.

## References

[1] Chen Z (2011). China Kadoorie Biobank of 0.5 million people: survey methods, baseline characteristics and long-term follow-up. Int. J. Epidemiol..

[2] Al Kuwari H (2015). The Qatar Biobank: background and methods. BMC Public Health.

[3] Kuriyama S (2016). The Tohoku Medical Megabank Project: design and mission. J. Epidemiol..

[4] Bycroft C (2018). The UK Biobank resource with deep phenotyping and genomic data. Nature.

[5] All of Us Research Program Investigators. The “All of Us” research program. *N. Eng. J. Med.* **381**, 668–676 (2019). 10.1056/NEJMsr1809937 PMC8291101 31412182

[6] Tennessen JA (2012). Evolution and functional impact of rare coding variation from deep sequencing of human exomes. Science.

[7] Lek M (2016). Analysis of protein-coding genetic variation in 60,706 humans. Nature.

[8] Genomes Project, C. (2015). A global reference for human genetic variation. Nature.

[9] Bergstrom A (2020). Insights into human genetic variation and population history from 929 diverse genomes. Science.

[10] Ahmad M (2017). Inclusion of population-specific reference panel from India to the 1000 genomes phase 3 panel improves imputation accuracy. Sci. Rep..

[11] Belsare S (2019). Evaluating the quality of the 1000 genomes project data. BMC Genomics.

[12] Huang L (2009). Genotype-imputation accuracy across worldwide human populations. Am. J. Hum. Genet..

[13] Conrad DF (2006). A worldwide survey of haplotype variation and linkage disequilibrium in the human genome. Nat. Genet..

[14] Wall JD, Pritchard JK (2003). Haplotype blocks and linkage disequilibrium in the human genome. Nat. Rev. Genet..

[15] Chen CH (2016). Population structure of Han Chinese in the modern Taiwanese population based on 10,000 participants in the Taiwan Biobank project. Hum. Mol. Genet..

[16] Hoffmann TJ (2011). Design and coverage of high throughput genotyping arrays optimized for individuals of East Asian, African American, and Latino race/ethnicity using imputation and a novel hybrid SNP selection algorithm. Genomics.

[17] Chen J (2009). Genetic structure of the Han Chinese population revealed by genome-wide SNP variation. Am. J. Hum. Genet..

[18] Xu S (2009). Genomic dissection of population substructure of Han Chinese and its implication in association studies. Am. J. Hum. Genet..

[19] Gao F, Keinan A (2016). Explosive genetic evidence for explosive human population growth. Curr. Opin. Genet. Dev..

[20] Gao F, Keinan A (2016). Inference of super-exponential human population growth via efficient computation of the site frequency spectrum for generalized models. Genetics.

[21] Bhaskar A, Wang YX, Song YS (2015). Efficient inference of population size histories and locus-specific mutation rates from large-sample genomic variation data. Genome Res..

[22] Vernot B (2016). Excavating Neandertal and Denisovan DNA from the genomes of Melanesian individuals. Science.

[23] Rutten JW (2019). The effect of NOTCH3 pathogenic variant position on CADASIL disease severity: NOTCH3 EGFr 1-6 pathogenic variant are associated with a more severe phenotype and lower survival compared with EGFr 7-34 pathogenic variant. Genet. Med..

[24] Hsieh FI, Chiou HY (2014). Stroke: morbidity, risk factors, and care in taiwan. J. Stroke.

[25] Lee YC, Chung CP, Chang MH, Wang SJ, Liao YC (2020). NOTCH3 cysteine-altering variant is an important risk factor for stroke in the Taiwanese population. Neurology.

[26] Rebours V, Levy P, Ruszniewski P (2012). An overview of hereditary pancreatitis. Dig. Liver Dis..

[27] GenomeAsia KC (2019). The GenomeAsia 100K Project enables genetic discoveries across Asia. Nature.

[28] Bachtiar M, Lee CGL (2013). Genetics of population differences in drug response. Curr. Genet. Med. Rep..

[29] Iyer L (2002). UGT1A1*28 polymorphism as a determinant of irinotecan disposition and toxicity. Pharmacogenomics J..

[30] Rauchschwalbe SK, Zuhlsdorf MT, Schuhly U, Kuhlmann J (2002). Predicting the risk of sporadic elevated bilirubin levels and diagnosing Gilbert’s syndrome by genotyping UGT1A1*28 promoter polymorphism. Int. J. Clin. Pharm. Ther..

[31] Sun W (2015). ABO blood types and cancer risk—a cohort study of 339,432 subjects in Taiwan. Cancer Epidemiol..

[32] Groot HE (2020). Genetically determined ABO blood group and its associations with health and disease. Arterioscler Thromb. Vasc. Biol..

[33] Zu BL, You GL, Fu QH, Wang J (2017). Association between ABO Blood Group and Risk of Congenital Heart Disease: a 6-year large cohort study. Sci. Rep..

[34] Dubinski D (2018). The clinical relevance of ABO blood type in 100 patients with acute subdural hematoma. PLoS ONE.

[35] Popejoy AB, Fullerton SM (2016). Genomics is failing on diversity. Nature.

[36] Sirugo G, Williams SM, Tishkoff SA (2019). The missing diversity in human genetic studies. Cell.

[37] Phillips EJ (2018). Clinical pharmacogenetics implementation consortium guideline for HLA genotype and use of carbamazepine and oxcarbazepine: 2017 update. Clin. Pharm. Ther..

[38] Martin MA (2012). Clinical pharmacogenetics implementation consortium guidelines for HLA-B genotype and abacavir dosing. Clin. Pharm. Ther..

[39] Saito Y (2016). Clinical Pharmacogenetics Implementation Consortium (CPIC) guidelines for human leukocyte antigen B (HLA-B) genotype and allopurinol dosing: 2015 update. Clin. Pharm. Ther..

[40] Liao YC (2015). Characterization of CADASIL among the Han Chinese in Taiwan: distinct genotypic and phenotypic profiles. PLoS ONE.

[41] Tang SC (2019). Prevalence and clinical characteristics of stroke patients with p.R544C NOTCH3 mutation in Taiwan. Ann. Clin. Transl. Neurol..

[42] Pan WH (2006). Han Chinese cell and genome bank in Taiwan: purpose, design and ethical considerations. Hum. Hered..

[43] Kidd JR (2011). Analyses of a set of 128 ancestry informative single-nucleotide polymorphisms in a global set of 119 population samples. Investig. Genet..

[44] Welter D (2014). The NHGRI GWAS Catalog, a curated resource of SNP-trait associations. Nucleic Acids Res..

[45] Li, H. Aligning sequence reads, clone sequences and assembly contigs with BWA-MEM. Preprint at https://arxiv.org/abs/1303.3997 (2013).

[46] Poplin, R. et al. Scaling accurate genetic variant discovery to tens of thousands of samples. Preprint at *bioRxiv*10.1101/201178 (2018).

[47] Delaneau O, Zagury JF, Marchini J (2013). Improved whole-chromosome phasing for disease and population genetic studies. Nat. Methods.

[48] Howie BN, Donnelly P, Marchini J (2009). A flexible and accurate genotype imputation method for the next generation of genome-wide association studies. PLoS Genet..

[49] Zheng X (2014). HIBAG-HLA genotype imputation with attribute bagging. Pharmacogenomics J..

[50] McLachlan S (2016). Replication and characterization of association between ABO SNPs and red blood cell traits by meta-analysis in Europeans. PLoS ONE.

