# Supplementary material for: Genetic profiles of 103,106 individuals in the Taiwan Biobank provide insights into the health and history of Han Chinese
Source: NPJ Genom Med. 2021 Feb 11;6:10. doi: 10.1038/s41525-021-00178-9 (PMC7878858; doi:10.1038/s41525-021-00178-9)
Supplement: Supplementary file 1 — Supplementary Information [file 41525_2021_178_MOESM1_ESM.pdf]

Supplemental Text

CNV Analysis

To assess the performance TWBv2 probes for known CNV analysis, we genotyped 96 individuals with SNP6.0 array data obtained previously for comparison. Of these, 31 were patients with confirmed chromosomal aberrations, 4 were healthy controls with induced genomic alterations during cell culture, and the remaining 61 were healthy controls without apparent chromosomal abnormalities. The CNVs detected by TWBv2 were compared to the ones that were previously identified using the ThermoFisher SNP6.0 array for the same individuals in three sets of analyses. First, we examined whether the known chromosomal aberrations from the 31 individuals based on SNP6.0 array analysis could be detected by TWBv2 array. Forty out of the 41 chromosomal aberrations (ranging from 108 kb to 26 Mb in length) previously found in these individuals by the SNP6.0 array were successfully genotyped on the TWBv2 array (Table S4). The one (615 kb) region not detected by the TWBv2 array was located at the telomere of Chr1p and was split into multiple pieces in the GRCh38/hg38 genome assembly. Second, we examined whether the genomic alterations in the EBV-transformed cells could be detected by the TWBv2 array. Because these genomic alterations were induced during cell culturing, the abnormalities were somatic and could be mosaic. Three chromosomal regions with gain or loss signals were clearly detected by TWBv2 (Figure S5a-S5c). However, the regions were divided into several segments because intensities of some SNPs did not pass the threshold for CNV calls. Third, we assessed the performance of the TWBv2 array in identifying regions of copy-neutral loss-of-heterozygosity (LOH). Applying the analytical approach we developed to detect LOH or CNV based on variation of B allele frequencies (BAF) over a region, we were able to identify samples consist of a mixture of cells with and without LOH (Figure S5d). These results show that the TWBv2 array can be used to detect germ-line as well as somatic genomic aberrations including deletions, duplications, and LOH.

Supplemental methods

Methods for CNV Analysis

CNV analysis and LOH analysis of the 96 test samples were carried out using Axiom Processing Tool v.2.11.1 (Thermo Fisher) with default setting. All the samples passed quality control and were included for reference set establishment. DNA segments with copy number state other than 2, probe numbers at least 20, and length at least 20 kb were selected for further analysis. The target chromosomal aberrant regions reported by SNP6.0 have been liftover from NCBI36/hg18 to GRCh38/hg38 for comparison. Among the forty-one target regions, four were failed to be converted to new version of human genome assembly because the regions were split in GRh38/hg38 assembly. Consistency between the target regions and CNV segments detected by TWB2.0 was defined as a CNV segment covering at least 50% of a given target region from the same individual. When there was more than one CNV segment overlapped with a target region, the flanking CNV segments were merged for coverage analysis.

Supplemental figures

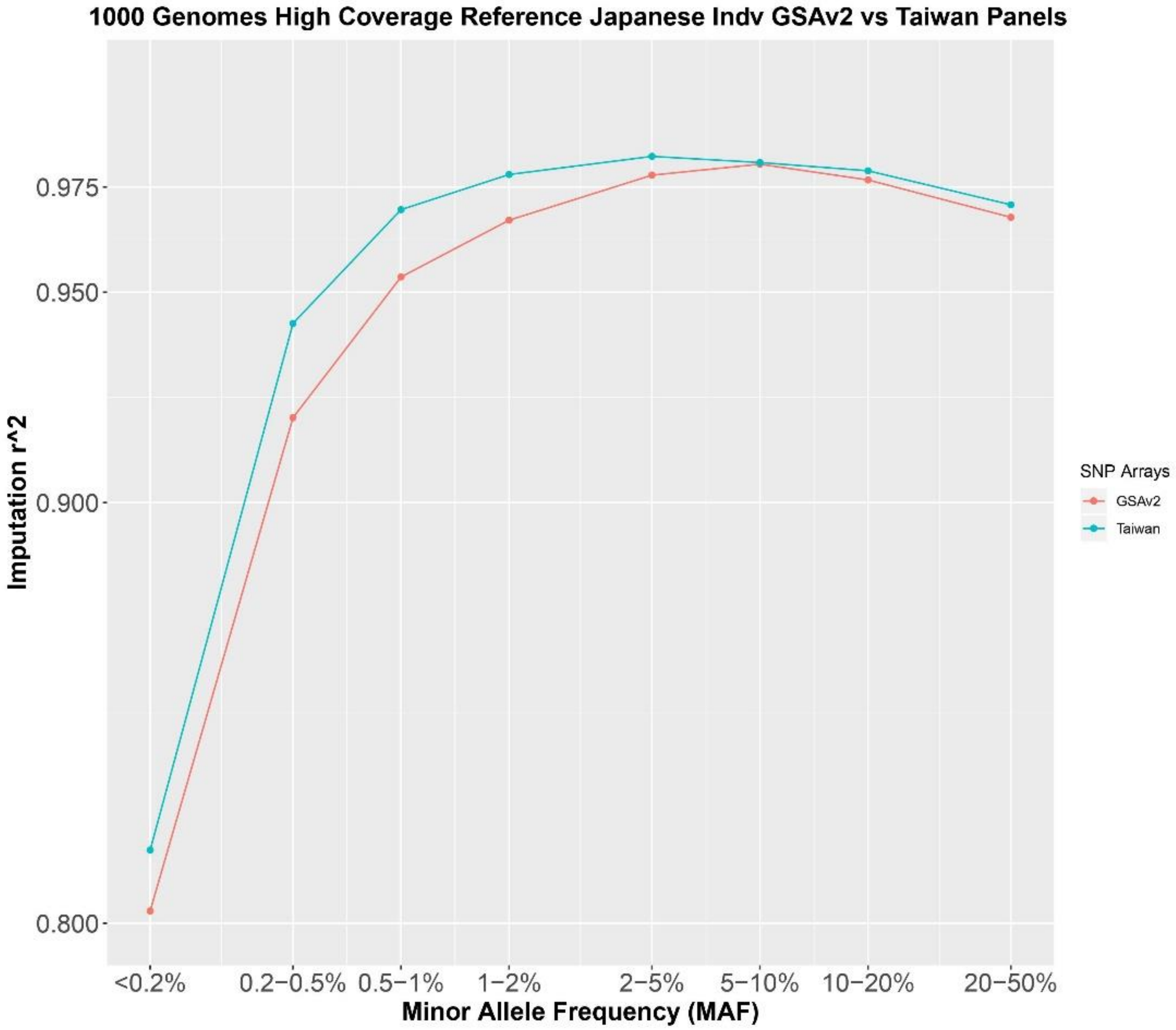

Supplementary Figure 1. Comparison of imputation accuracy (as measured by r^2 between imputed and actual genotypes), stratified by MAF, using the TWBv2 array versus the GSA2 array. For both, the reference panel is the 1000 Genomes Project high-coverage data and the test samples are the Japanese samples from the GenomeAsia 100K Consortium study.

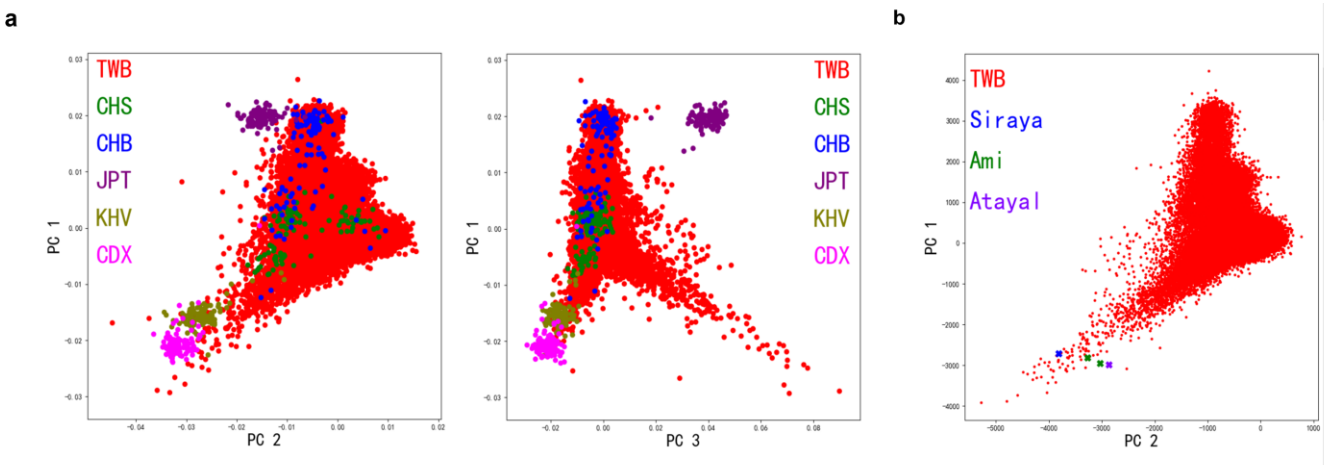

Supplementary Figure 2. PCA of the TWB participants with samples from 1000 genome projects and SGDP. (a) PCA plots for from 1000 genome project samples and TWB (CDX:Chinese Dai in Xishuangbanna, China; CHB: Han Chinese in Beijing, China; CHS: Southern Han Chinese; JPT: Japanese in Tokyo, Japan; KHV: Kinh in Ho Chi Minh City, Vietnam; TWB: Taiwan Biobank Participants). (b) PCA analysis showed that a Siraya in the TWB cohort was clustered with three Taiwanese indigenous people (2 Amei and 1 Atayal) from SGPD. PC1 and PC2 are the first two components derived from the PCA.

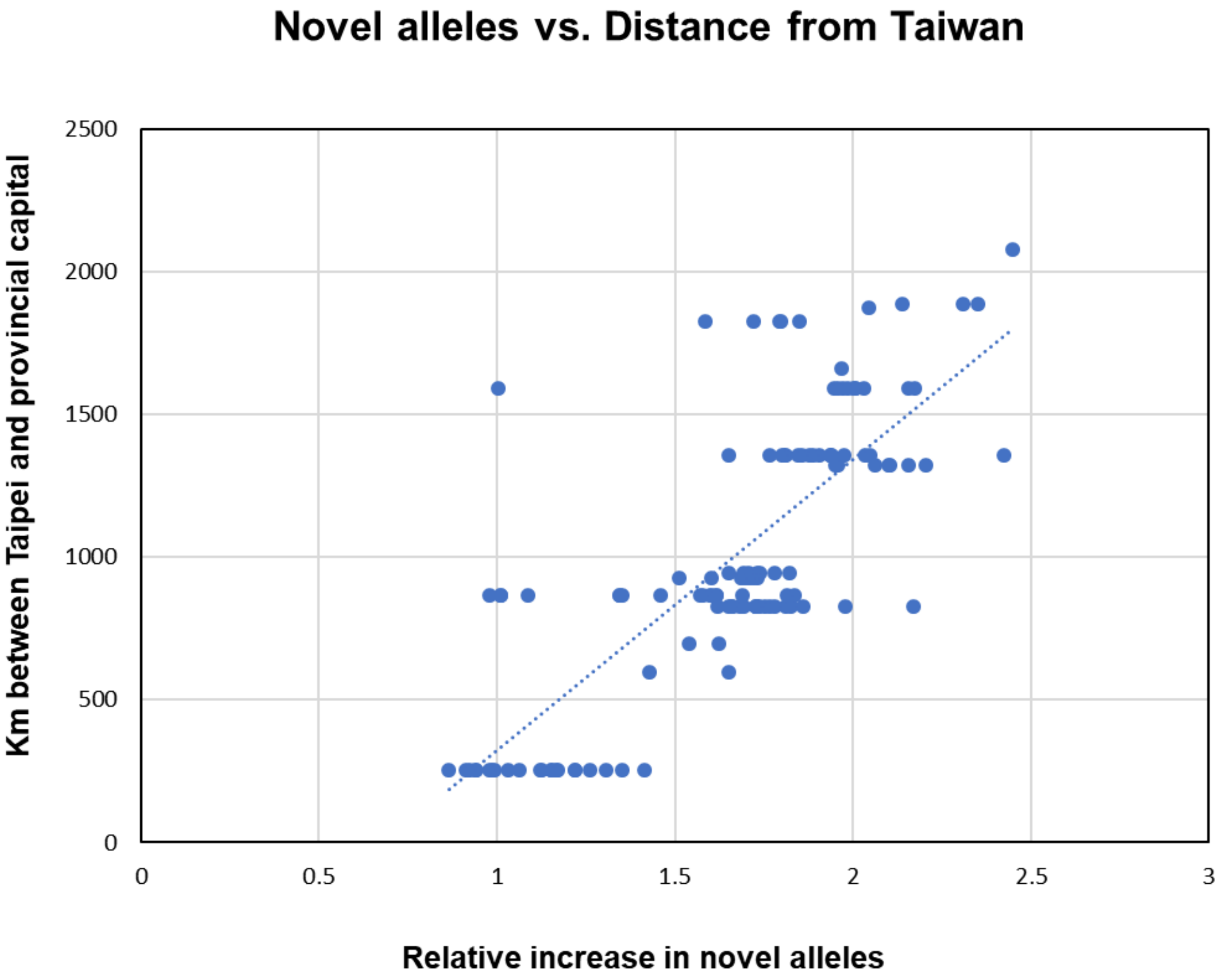

Supplementary Figure 3. Scatterplot of relative increase in novel alleles versus distance between a sample's provincial capital and Taipei.

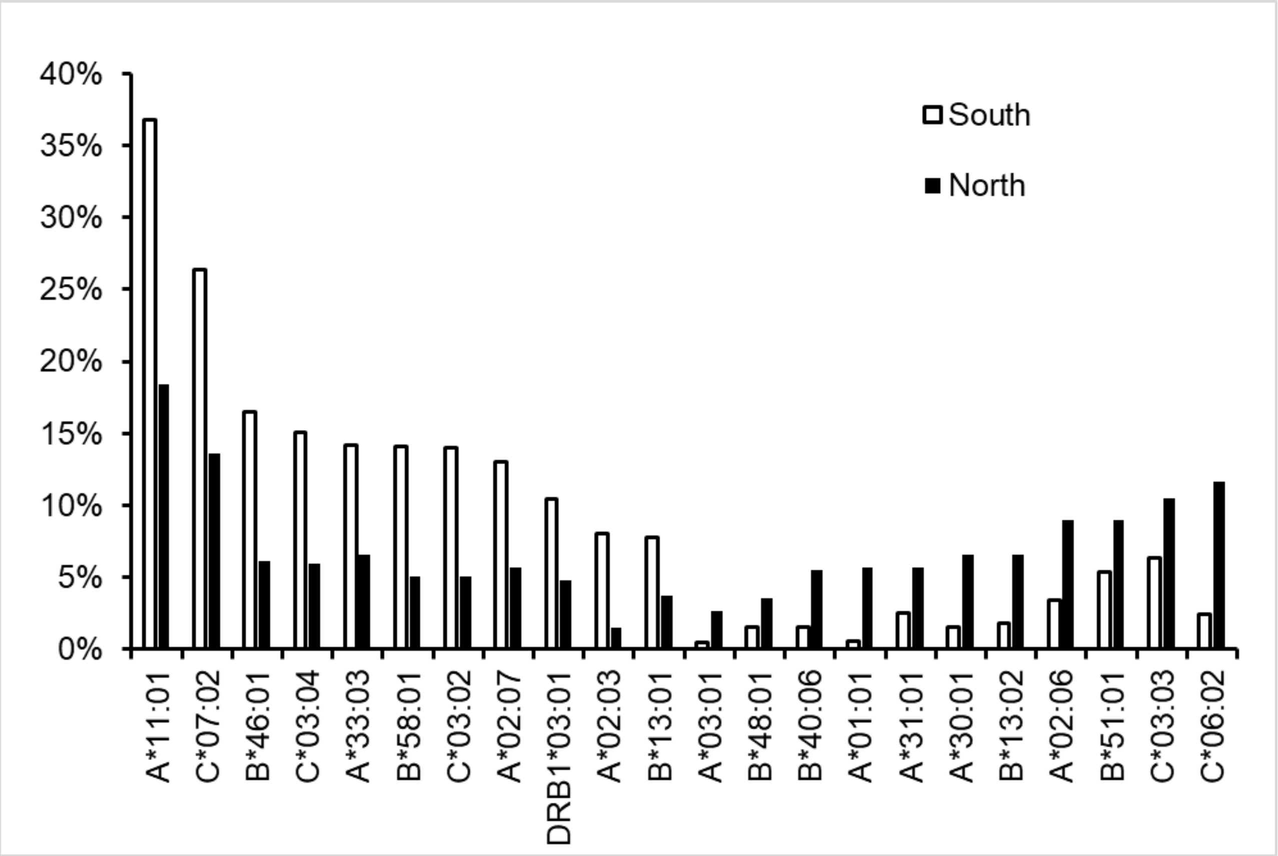

Supplementary Figure 4. Allele frequency and distribution of imputed high-resolution HLA types in TWB participants based on geographic origins.

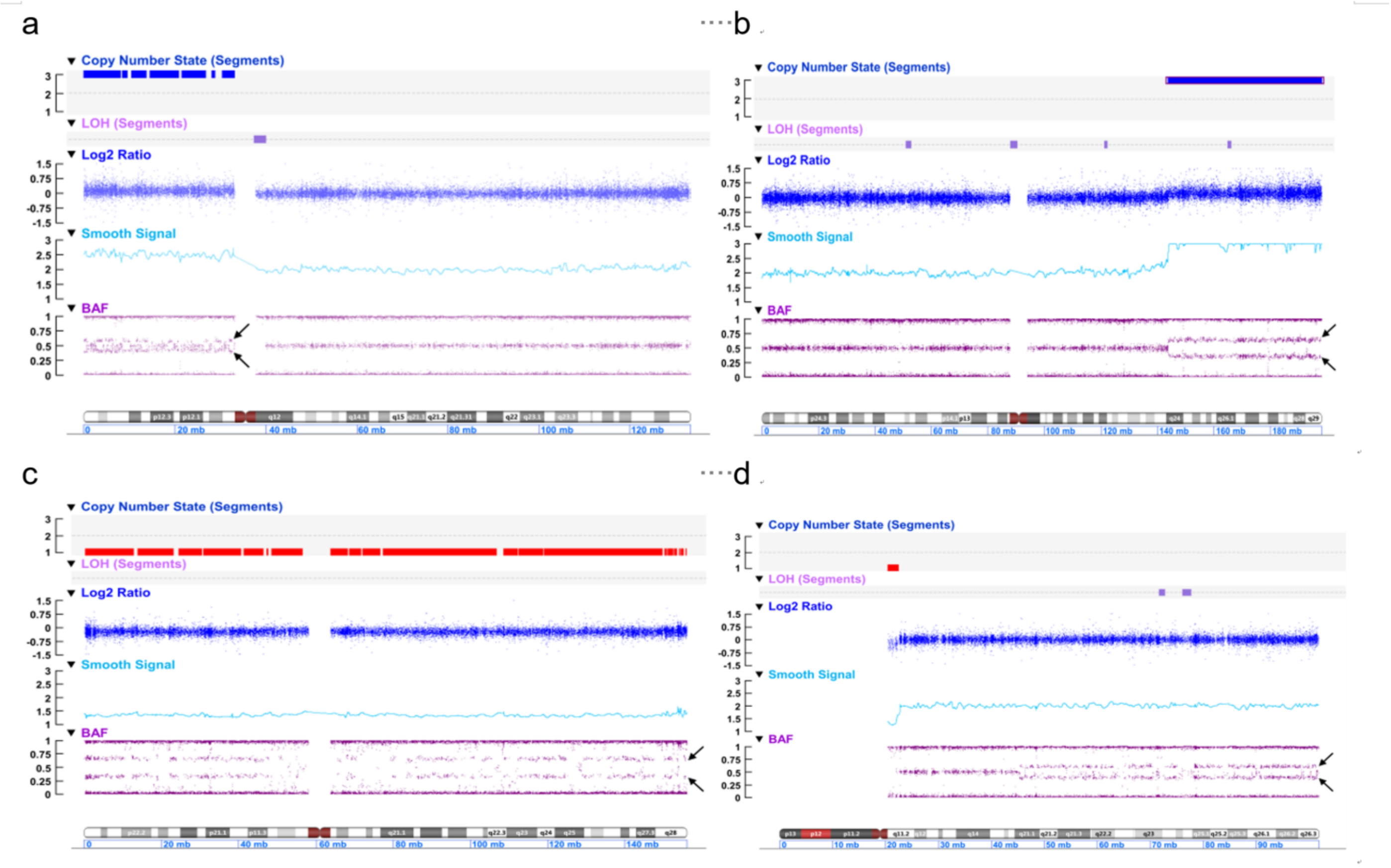

Supplementary Figure 5. Mosaic chromosomal abnormalities of EBV-transformed B cells detected by TWB2.0 array. (a). Mild mosaic duplication of Chr12p is detected based on the presence of several segments with duplication signals and abnormal B-allele frequency (BAF) within the regions. (b). Severe mosaic duplication of Chr3q24-q29 is detected based on the presence of one segment with duplication signals. (c). Moderate mosaic deletion of ChrX is detected based on the presence of multiple segments with deletion signals and obvious abnormal BAF. (d). Mild mosaic copy-neutral LOH is detected based on the presence of abnormal BAF. Arrows indicate SNPs with abnormal BAF.

Supplemental tables

Supplementary Table 1. Content of TWBv2 array

Category	Variants
GWAS grid	~415,000
CNV	~57,000
X chromosome	~6,800
Y chromosome	~14,000
Mitochondrial	~800
Health status-associated variants	~2,000
GWAS catalog	~40,000
ClinVar	~56,000
ACMG	~16,000
Pharmacogenetics/ADME	~4,100
HLA	~9000
KIR	~1400
Pathogenic or likely pathogenic	~44,000
Autosomal dominant	~2700
Autosomal recessive	~4000

Supplementary Table 2. Targeted CNV regions

Chrom	Start_position	End_position	Gene	Chrom	Start_position	End_position	Gene
1	16935740	16968705		5	1685479	2197089	
1	145809392	145829793		5	122560082	123202432	
1	145917713	145927678	RBM8A	5	142628184	142960105	
1	146147883	148893213		5	148002330	148586891	
1	152436463	152469824		5	172276341	172744530	
1	174833002	174833691		6	113434010	116771846	
1	244716010	245697402	HNRNPU	6	161347419	162259273	PARK2
1	244716010	245697402		6	166409363	166906451	RPS6KA2
1	247757461	247758406	OR1C1	7	1164205	1598860	
1	248238928	248239864	OR2M4	7	15843927	16300363	
1	248592829	248593768	OR2T10	7	39097461	39506174	
2	15598026	15795714		7	65373855	65401085	
2	49918504	51032536	NRXN1	7	73328061	74760692	STX1A
2	74630228	75097478		7	73328061	74760692	GTF2I
2	90082547	90095707		7	93743299	93772662	
2	91613447	91948753		7	105036075	105458896	
2	97195895	97200085		7	116725042	116803220	
2	162696789	163351864		7	119673168	120603257	
2	201652291	201663822		7	127626828	128029951	
2	214995983	215467926		7	145367649	146253362	
2	238244037	238290102	PER2	7	156994532	159327018	VIPR2
2	239048167	239401654	HDAC4	8	612629	1209817	
2	241849880	241858908	PDCD1	8	2935352	4994972	CSMD1
3	1092575	1404217	CNTN6	8	6406591	6648505	MCPH1
3	19242123	19803162		8	10764960	10839899	PINX1
3	75590344	75881769		8	12685212	12729881	
3	100128858	100580485		8	13420325	13984283	
3	130088069	130196115		8	16107877	16567490	MSR1
3	195746764	195812277	MUC4	8	18527300	19084730	PSD3
3	196018732	197628732	DLG1	8	74880842	75321984	
3	196018732	197628732	TM4SF19	8	96158782	96524921	
3	196018732	197628732		8	99013266	99877580	
4	2741647	2756376	TNIP2	9	841690	969090	
4	3884069	4188833		9	4533853	4538277	
4	9484428	9742967		9	22190563	22988893	
4	44007184	44584699		9	24466997	24495716	
4	67612651	67701179	UBA6	9	122660145	123184806	
4	68506273	68544493		9	135702184	135795508	KCNT1

Chrom	Start_position	End_position	Gene	Chrom	Start_position	End_position	Gene
9	137138389	137168762	GRIN1	16	29506378	30191178	SEZ6L2
9	137618962	137870016	EHMT1	16	29506378	30191178	KCTD13
9	137877788	138124624	CACNA1B	16	29506378	30191178	MAPK3
10	34381600	35039494		16	30698208	30741409	SRCAP
10	49107035	49671832		16	31106106	31112791	BCKDK
10	126450084	126909264		16	31549193	31645257	
11	4545190	4546144	OR52M1	16	34061880	34208804	
11	20004327	20966564		16	34962968	35268851	
11	31240619	31688096		16	75528529	75535247	CHST5
12	6942977	6946003	C12orf57	16	75536743	75556286	TMEM231
12	7129697	7158945	CLSTN3	16	76277216	76560750	CNTNAP4
12	8225643	8384004		16	77247812	77435114	ADAMTS18
12	33599395	34379787		17	930550	1613186	YWHAE
12	128126688	128487159		17	2059838	2303775	SMG6
12	130094548	130645732		17	2384059	2401118	MNT
13	19837453	19863633		17	2593209	2685615	PAFAH1B1
14	21385193	21456126	CHD8	17	17494436	17496395	RASD1
14	22773221	22829820	SLC7A7	17	17681472	17811453	RAI1
14	23960089	24044696		17	21376196	21419872	KCNJ12
14	70688249	71233331		17	22667724	22688611	
14	98588943	98696797		17	39626739	39636626	PPP1R1B
15	22773062	22867050	NIPA1	17	79892322	80316385	
15	22773062	22867050	NIPA2	17	80977353	81538598	
15	22868385	23074431	CYFIP1	18	7079986	7563167	
15	22868385	23074431	TUBGCP5	18	47227732	47785629	
15	23643543	23647846	MAGEL2	18	55341734	55477638	
15	24823636	24978723	SNRPN	19	27525358	27564507	
15	25333727	25439056	UBE3A	20	12761990	13357690	
15	28464134	28828395		20	13995368	16053197	MACROD2
15	30840505	32166459	CHRNA7	20	24993133	25736933	
15	30840505	32166459	FAN1	20	59447284	60432815	
15	30840505	32166459	TRPM1	21	13084083	13221902	
15	32172522	32565269		21	21898323	22362794	
15	32853510	33253897		22	18157533	18190106	
16	1153240	1221772	CACNA1H	22	18912773	18936553	PRODH
16	2047464	2088720	TSC2	22	19179472	19291716	CLTCL1
16	2253115	2268412	RNPS1	22	19756702	19783222	TBX1
16	3725053	3880726	CREBBP	22	19783594	19854939	GNB1L
16	6019093	7713338	RBFOX1	22	20979461	20999038	LZTR1
16	28811178	29041178		22	21111546	21257048	

Chrom	Start_position	End_position	Gene	Chrom	Start_position	End_position	Gene
22	21562852	21953147		23	150766335	150898816	CD99L2
22	48992889	50750066	CHKB	23	152166233	152451359	GABRA3
22	48992889	50750066	MAPK12	23	152638182	152657534	GABRQ
22	48992889	50750066	MAPK8IP2	23	153688098	153696593	SLC6A8
22	48992889	50750066	SBF1	23	153704720	156004181	HCFC1
22	48992889	50750066	SHANK3	23	153704720	156004181	MECP2
22	50648836	50796015		23	153704720	156004181	PDZD4
23	6022606	8177680		23	153704720	156004181	PLXNA3
23	57357614	58411744		23	153704720	156004181	RAB39B
23	68223324	68681109		23	153704720	156004181	RPL10
23	148500618	149000663	AFF2	23	153704720	156004181	TMLHE
23	149712024	149717267					

Supplementary Table 3. Concordance of genotypes by TWBv2 Array and Sequencing.

Minor Allele Frequency (%)	Number of Markers	Concordance Rate (S.D.)
0	110,497	99.6% (5.7)
0.1 - 1	47,631	99.8% (3.8)
1 - 5	204,981	99.9% (0.9)
5 - 10	73,263	99.8% (1.9)
10 - 20	77,125	99.8% (2.5)
29 - 30	54,953	99.7% (3.1)
30 - 40	47,449	99.6% (4.0)
40 - 50	44,707	99.4% (6.3)
Total	660,606	99.8% (3.5)

S.D.: standard deviation

Supplementary Table 4. CNV detection by 6.0 SNP array and TWBv2 SNP array.

			SNP 6.0				TWBv2			
Case ID	**Sex**	**Cytoband**	**Start**	**End**	**Size (bp)**	**Gain/ Loss**	**Start**	**End**	**Markers**	**Size (bp)**
**203897**	M	7p21.1	15843927	16300363	456436	Gain	15843802	16300709	79	456907
**203897**	M	12q24.32	128126688	128487159	360471	Gain	128119145	128487240	180	368095
**206973**	M	18q21.1	47227732	47785629	557897	Loss	47227670	47806193	131	578523
**215325**	F	5q35.1	172276341	172744530	468189	Loss	172275841	172745030	173	469189
**215325**	F	16q21	64086661	90221115	26134454	Gain	64069732	90215023	8775	26145291
**217294**	M	16p11.2	29506378	30178709	672331	Loss	29580435	30191213	404	610778
**218348**	M	16p13.3	10001	1543842	1533841	Loss	29043	1367092	645	1338049
**218867**	M	Xq28	153704720	156004181	2299461	Gain	153687598	156008488	1792	2320890
**220156**	F	1p36.33	Split in Hg38 Assembly			Loss	784860	1915143	420	1130283
**220156**	F	20p12.1	12761990	13357690	595700	Loss	12761490	13357786	188	596296
**225877**	M	16p11.2	29641167	30179356	538189	Loss	29580435	30159695	406	579260
**226996**	F	5q31.3	142628184	142960105	331921	Gain	142619401	143013997	160	394596
**226996**	F	5q33.1	148002330	148586891	584561	Gain	147998200	148587391	296	589191
**228205**	M	20p11.21	24993133	25736933	743800	Loss	24992611	25685798	137	693187
**228263**	F	2p24.3	15598026	15795714	197688	Gain	15597574	15796184	110	198610
**228432**	F	6q22.1	113620909	116771846	3150937	Gain	113603564	116771942	477	3168378
**228488**	F	22q11.21	Split in Hg38 Assembly			Loss	18889117	21097474	1108	2208357
**228838**	M	6q21	113434010	116321905	2887895	Loss	113433933	116310960	370	2877027
**229000**	F	11p15.1	20004327	20966564	962237	Gain	20015574	20970661	291	955087
**229602**	M	3q12.1	100128858	100580485	451627	Gain	100123613	100561159	80	437546
**229604**	M	1q44	245150172	245697402	547230	Gain	245150423	245697748	278	547325
**229928**	F	1q44	244716010	245263357	547347	Loss	244722626	245268001	269	545375
**230277**	F	5q23.2	122560082	123202432	642350	Gain	122559645	123202932	155	643287
**230667**	M	17q25.3	79892322	80316385	424063	Gain	79874333	80319145	374	444812
**231447**	M	2q24.2	162696789	163351864	655075	Gain	162696384	163351945	98	655561
**231450**	F	18q21.2	55341734	55477638	135904	Loss	55340922	55474417	74	133495
**231515**	M	14q32.2	98588943	98696797	107854	Loss	98588943	98691093	51	102150
**231515**	M	14q32.2	98993937	100426412	1432475	Loss	98984298	100427519	370	1443221
**232616**	M	8q22.1	96158782	96524921	366139	Loss	96178811	96515450	65	336639

**234495**	F	10q11.22	49107035	49671832	564797	Gain	49105576	49671832	334	566256
**234495**	F	21q21.1	21898323	22362794	464471	Gain	21897823	22363294	141	465471
**235423**	M	22q11.21	Split in Hg38 Assembly			Loss	18905062	18933784	72	28722
**235423**	M	22q11.21	20756002	21077265	321263	Loss	18948253	21098561	1039	2150308
**237718**	M	11p13	31240619	31688096	447477	Loss	31240618	31701967	85	461349
**237886**	F	7q36.3-qter	156994532	159327018	2332486	Gain	157011816	159329032	585	2317216
**237886**	F	10p14-pter	14061	7627087	7613026	Loss	73872	7627589	2678	7553717
**238985**	F	1p36.33	Split in Hg38 Assembly							
**238985**	F	1p36.33	690078	3827697	3137619	Loss	784860	3826707	1288	3041847
**238985**	F	1p36.32	4662167	5746289	1084122	Loss	4662604	5745451	441	1082847
**239225**	F	14q24.2	70688249	71233331	545082	Gain	70683704	71244908	168	561204
**239772**	F	2q35	214995983	215467926	471943	Gain	215007788	215467943	121	460155

Supplementary Table 5. Population frequencies of known disease susceptibility variants.

	Disease	Gene	Variant ID	Allele frequency
**Cancer susceptibility**	Adenomatous polyposis coli	APC	rs1554086805	0.11%
			rs1554087314	0.12%
	Breast-ovarian cancer, familial 1	BRCA1	rs886040243	0.11%
			rs80356891	0.12%
	Breast-ovarian cancer, familial 2	BRCA2	rs80359373	0.15%
	Hereditary cancer-predisposing syndrome	CHEK2	rs786202601	0.31%
			rs864622613	0.13%
		RAD51D	rs753862052	0.12%
	Juvenile polyposis syndrome	SMAD4	rs876660720	0.10%
	Li-Fraumeni syndrome 1	TP53	rs397516436	0.15%
	Lynch syndrome	MSH6	rs876661171	0.42%
	MYH-associated polyposis	MUTYH	rs762307622	0.11%
	Neuroblastoma	KIF1B	rs121908163	0.41%
	PTEN hamartoma tumor syndrome	PTEN	rs1554825615	0.10%
	Prostate cancer	EHBP1	rs721048	3.88%
**Autosomal dominant**	Cerebral autosomal dominant arteriopathy with subcortical infarcts and leukoencephalopathy type 1 (CADASIL)	NOTCH3	rs201118034	0.48%

	DFNA2 Nonsyndromic Hearing Loss	KCNQ4	rs80358273	0.62%
	Familial hypercholesterolemia	APOB	rs144467873	0.19%
		LDLR	rs730882109	0.18%
			rs749038326	0.09%
	Familial hypertrophic cardiomyopathy	TCAP	rs778568339	0.23%
	Hereditary pancreatitis	SPINK1	rs148954387	0.43%
		PRSS1	rs387906698	0.10%
	Maturity-onset diabetes of the young type 3	HNF1A	rs776793516	0.11%
	Spastic paraplegia 4	SPAST	rs864622162	0.14%
**Autosomal recessive**	Joubert Syndrome	CEP290	rs760540562	0.25%
	Meckel syndrome	MKS1	rs762668200	0.15%
	Primary Autosomal Recessive Microcephaly	MCPH1	rs541042265	0.13%
	Congenital hypothyroidism	TSHR	rs189261858	0.36%
	Total iodide organification defect	TPO	rs770781635	0.28%
	ABCA4-Related Disorders	ABCA4	rs752786160	0.17%
	Bestrophinopathy	BEST1	rs200277476	0.15%
	Cone-rod dystrophy and hearing loss 1	CEP78	rs745750156	0.08%
	Nonsyndromic hearing loss and deafness	GJB2	rs80338943	0.69%
			rs111033204	0.10%
	Pendred syndrome	SLC26A4	rs111033313	0.76%
			rs121908362	0.10%

Usher syndrome	USH2A	rs201527662	0.25%
beta Thalassemia	HBB	rs80356821	0.29%
Hereditary factor XI deficiency disease	F11	rs770505620	0.19%
Carnitine palmitoyltransferase II deficiency	CPT2	rs74315298	0.10%
Citrullinemia type II	SLC25A13	rs80338720	0.55%
		rs80338717	0.42%
Congenital Disorder of Glycosylation 1a	PMM2	rs150719105	0.20%
Gaucher disease	GBA	rs76539814	0.11%
Glutaric aciduria 1	GCDH	rs199999619	0.19%
Glycogen storage disease 1A	G6PC	rs1801176	0.10%
Mehylmalonic aciduria	MMUT	rs753288303	0.10%
Mucolipidosis type III	GNPTAB	rs397507447	0.13%
		rs200646278	0.09%
Phenylketonuria	PAH	rs76687508	0.24%
Pompe disease	GAA	rs28940868	0.19%
Primary carnitine deficiency	SLC22A5	rs60376624	0.28%
		rs121908893	0.18%
PTPS deficiency	PTS	rs104894275	0.14%
Sialidosis	NEU1	rs398123392	0.18%
Sitosterolemia	ABCG5	rs119480069	0.33%
Waardenburg syndrome	EDNRB	rs781214034	0.20%

	Wilson disease	ATP7B	rs541208827	0.30%
			rs750019452	0.28%
			rs28942074	0.19%
			rs779323689	0.12%
	Hereditary spastic paraplegia 5	CYP7B1	rs200737038	0.42%
	Hereditary spastic paraplegia 15	ZFYVE26	rs769329153	0.11%
	Krabbe Disease	GALC	rs138577661	0.84%
	Limb-girdle muscular dystrophy 2A	TRAPPC11	rs397509417	0.10%
	Autoimmune polyendocrinopathy syndrome type I	AIRE	rs199612115	0.16%
	Bardet-Biedl syndrome	BBS2	rs773862084	0.19%
	Cystic fibrosis	CFTR	rs115545701	0.11%
	Nagashima-type palmoplantar keratosis	SERPINB7	rs142859678	0.72%
**X-linked recessive**	G6PD deficiency	G6PD	rs72554664	0.69%
			rs137852331	0.27%
			rs137852340	0.21%
			rs137852342	0.18%
			rs137852341	0.17%
**Disease risk**	Alzheimer's disease	APOE	rs429358	8.65%
			rs7412	7.36%
	Hyperuricemia	ABCG2	rs2231142	31.40%

Alcohol-flushing reaction	ALDH2	rs671	28.00%
Ankylosing spondylitis	HLA-B*27:04		3.50%
Grave's disease	HLA-B*46:01		13.30%

Supplementary Table 6. Variants associated with drug metabolism and adverse drug reactions.

Biomarker	Variant	Enzyme activity/ ADR relationship	Allele	Drugs	Allele frequency
CYP2B6	rs3745274	Decreased or no function		Efavirenz	18.8%
CYP2C9	rs1057910	Decreased function	*3	Celecoxib	3.3%
CYP2C19	rs4244285	No function	*2	Citalopram	31.9%
	rs4986893	No function	*3	Clobazam	5.2%
	rs72552267	No function	*6	Clopidogrel	0.2%
CYP3A5	rs776746	No function	*3	Tacrolimus	71.6%
HLA-A*3101		Risk		Carbamazepine	2.1%
HLA-B*1502		Risk		Carbamazepine	4.3%
HLA-B*5701		Risk		Abacavir	0.4%
HLA-B*5801		Risk		Allopurinol	10.8%
IFNL3/IL28B	rs12979860	Unfavorable response genotype		Peginterferon Alfa	5.9%
MT-RNR1	rs28358569	Risk	m. 827 A>G	Amikacin	4.4%
	rs267606618	Risk	m. 1095 T>C	Gentamicin	0.2%
	rs267606617	Risk	m. 1555 A>G	Neomycin	0.2%
NAT2	rs1801280	Decreased function		Isoniazid	26.4%
	rs1799930	Decreased function		Sulfasalazine	16.0%
	rs1799931	Decreased function		Co-trimoxazole	4.1%
SLCO1B1	rs4149056	Decreased function	*5,15,17	Simvastatin	11.2%
TPMT	rs1142345	No function	*3C	Azathioprine	1.5%
UGT1A1	rs4148323	Decreased function	*6	Atazanavir	15.1%
	rs35350960	Decreased function	*27	Irinotecan	2.1%
	rs887829	Decreased function	*80+*28	Nilotinib	12.1%
VKORC1	rs9923231	Decreased warfarin dose		Warfarin	89.2%

Supplementary Table 7. Consistency of imputed HLA types in parent-child pairs.

	ABO	HLA-A	HLA-B	HLA-C	HLA-DPB1	HLA-DQB1	HLA-DRB1
Consistency	99.9%	97.8%	97.4%	98.9%	99.2%	99.7%	97.5%

Supplementary Table 8. Accuracy of estimated two-field HLA types with a posterior probability call threshold of 0.

Data source	A	B	C	DRB1	DQB1	DPB1
TWBv1 (imputed)	97.5%	97.7%	96.6%	97.0%	99.8%	98.3%
TWBv2	97.4%	95.3%	98.1%	91.4%	99.4%	96.1%

Supplementary Table 9. 2831 rare genetic disease genes with variants on the TWBv2 array.

A2ML1	ACVRL1	AICDA	AMN	AQP5	ATM	BBS2	C12orf57
AAAS	ADA	AIP	AMPD2	ARCN1	ATP13A2	BBS4	C12orf65
AARS1	ADA2	AIPL1	AMPD3	ARFGEF1	ATP1A1	BBS5	C15orf41
AASS	ADAM9	AIRE	ANAPC15	ARFGEF2	ATP1A3	BBS7	C17orf107
ABAT	ADAMTS10	AK1	ANGPTL3	ARG1	ATP2A1	BBS9	C19orf12
ABCA1	ADAMTS13	AK2	ANK1	ARHGAP11A -SCG5	ATP2A2	BCHE	C1QA
ABCA12	ADAMTS17	AKAP10	ANK2	ARHGAP26	ATP5F1A	BCKDHA	C1QTNF3 -AMACR
ABCA4	ADAMTS18	AKAP9	ANK3	ARHGAP31	ATP6V0A2	BCKDHB	C1S
ABCB11	ADAMTS2	AKR1D1	ANKH	ARHGDIA	ATP6V0A4	BCS1L	C2
ABCB4	ADAMTSL2	AKT1	ANKLE2	ARHGEF10	ATP6V1B2	BEST1	C2CD3
ABCB6	ADAMTSL4	AKT2	ANKRD11	ARID1A	ATP7B	BHLHA9	C3
ABCC2	ADAR	AKT3	ANKRD26	ARID1B	ATP8B1	BICC1	C5
ABCC8	ADCY1	ALAD	ANKS6	ARID2	ATPAF2	BICD2	C6
ABCC9	ADCY6	ALDH18A1	ANLN	ARL13B	ATR	BIN1	C7
ABCD3	ADD3	ALDH1A3	ANO10	ARL14EP	AUH	BLK	C8B
ABCD4	ADGRE2	ALDH3A2	ANO3	ARL16	AURKC	BLM	C8orf37
ABCG8	ADGRG1	ALDH5A1	ANO5	ARL2BP	AUTS2	BLOC1S6	C9
ABHD12	ADGRG6	ALDH6A1	ANTXR1	ARL6	AVP	BMP1	CA1
ABHD5	ADGRV1	ALDH7A1	ANTXR2	ARL6IP1	AXIN2	BMP4	CA12
ACAD8	ADK	ALDOA	AP2S1	ARMC4	B2M	BMPER	CA2
ACAD9	ADNP	ALDOB	AP3B1	ARMC5	B3GALNT2	BMPR1A	CA4
ACADM	ADSL	ALG1	AP3B2	ARSA	B3GALT6	BMPR2	CA5A
ACADS	ADSS1	ALG11	AP3D1	ARSB	B3GAT3	BMS1	CA8
ACADSB	AFF4	ALG12	AP4B1	ARV1	B3GLCT	BOLA3	CABP2
ACADVL	AFG3L2	ALG14	AP4M1	ASAH1	B3GNT6	BPGM	CABP4
ACAN	AFP	ALG2	AP4S1	ASB10	B4GALNT1	BRAF	CACNA1A
ACAT1	AGA	ALG6	AP5Z1	ASCC1	B4GALT7	BRAT1	CACNA1C
ACD	AGBL1	ALG8	APC	ASL	B4GAT1	BRCA1	CACNA1E
ACE	AGBL5	ALG9	APOA1	ASNS	B9D1	BRCA2	CACNA1H
ACO2	AGK	ALK	APOA1-AS	ASPA	B9D2	BRD7	CACNB2
ACOX1	AGL	ALMS1	APOA5	ASPH	BAAT	BRF1	CACNB4
ACOX2	AGPAT2	ALOX12B	APOB	ASPM	BAG3	BRIP1	CAD
ACP4	AGPS	ALOXE3	APOC2	ASS1	BANF1	BSCL2	CALM1
ACP5	AGRN	ALPL	APOC3	ASXL1	BAP1	BSND	CALM2
ACTA1	AGT	ALS2	APOE	ASXL2	BARD1	BTD	CALR
ACTA2	AGXT	ALX1	APOL1	ASXL3	BAX	BVES	CANT1
ACTB	AGXT2	ALX3	APPL1	ATF6	BBIP1	C10orf105	CAPN1
ACTG1	AHCY	ALX4	APRT	ATIC	BBS1	C11orf65	CAPN3
ACTN2	AHDC1	AMH	APTX	ATL1	BBS10	C12orf4	CAPN5
ACVR2B	AHI1	AMHR2	AQP1	ATL3	BBS12	C12orf43	CARD10

CARD11	CD164	CEACAM16	CHKB	CNTN2	COQ4	CSRP3	CYP7B1
CARD14	CD19	CEL	CHMP2B	CNTN4	COQ7	CST3	D2HGDH
CARD9	CD207	CENPE	CHN1	CNTNAP1	COQ8A	CSTA	DAG1
CARMIL2	CD247	CENPF	CHRM3	CNTNAP2	COQ8B	CSTB	DARS2
CARTPT	CD27	CENPJ	CHRNA1	COA3	COQ9	CTC1	DBH
CASP10	CD36	CEP104	CHRNA2	COA6	CORIN	CTCF	DBH-AS1
CASP8	CD3D	CEP120	CHRNA4	COA8	CORO1A	CTH	DBT
CASQ2	CD3E	CEP135	CHRNB1	COASY	COX10	CTHRC1	DCAF8
CASR	CD3G	CEP152	CHRNB2	COG4	COX14	CTLA4	DCC
CAST	CD40	CEP164	CHRND	COG8	COX15	CTNNA1	DCDC2
CASTOR3	CD46	CEP290	CHRNE	COL10A1	COX20	CTNNA3	DCHS1
CAT	CD59	CEP41	CHST14	COL11A1	COX6A1	CTNNB1	DCLRE1C
CATSPER1	CD79B	CEP55	CHST3	COL11A2	COX6B1	CTNS	DCN
CAV1	CD8A	CEP57	CHST6	COL12A1	COX8A	CTPS1	DCPS
CAV3	CDAN1	CEP63	CHSY1	COL13A1	CPAMD8	CTSA	DCTN1
CBL	CDC14A	CEP78	CHUK	COL17A1	CPLANE1	CTSC	DCXR
CBLIF	CDC42	CEP83	CIB2	COL1A1	CPT1A	CTSD	DDB2
CBS	CDC45	CERKL	CIDEC	COL1A2	CPT1C	CTSF	DDC
CBX2	CDC6	CERS3	CIITA	COL25A1	CPT2	CTSK	DDC-AS1
CC2D1A	CDC73	CETP	CIT	COL27A1	CR2	CUBN	DDHD2
CC2D2A	CDCA3	CFAP298	CKAP2L	COL2A1	CRADD	CUL3	DDOST
CCBE1	CDCA7	CFAP53	CLCN1	COL3A1	CRB1	CUL7	DDR2
CCDC114	CDCA7L	CFB	CLCN2	COL4A1	CRB2	CWF19L1	DDX11
CCDC115	CDH1	CFC1	CLCNKA	COL4A2	CREBBP	CXCR4	DDX41
CCDC151	CDH23	CFD	CLCNKB	COL4A2-AS1	CRPPA	CYB5A	DDX58
CCDC174	CDH23-AS1	CFH	CLDN14	COL4A4	CRPPA-AS1	CYB5R3	DDX59
CCDC39	CDH3	CFHR5	CLDN19	COL5A1	CRTAP	CYBA	DEAF1
CCDC40	CDHR1	CFI	CLMP	COL5A2	CRX	CYC1	DENND3
CCDC47	CDK4	CFL2	CLN3	COL6A1	CRYAA	CYCS	DENND5A
CCDC65	CDK5	CFTR	CLN5	COL6A2	CRYAB	CYLD	DEPDC5
CCDC88A	CDK5RAP2	CFTR-AS1	CLN6	COL6A3	CRYBB1	CYP11A1	DES
CCDC88C	CDK6	CHAMP1	CLN8	COL7A1	CRYBB2	CYP11B1	DGAT1
CCM2	CDKAL1	CHAT	CLPB	COL8A2	CRYBB3	CYP11B2	DGKE
CCN6	CDKN1B	CHCHD10	CLPP	COL9A1	CRYGA	CYP17A1	DGUOK
CCND1	CDKN1C	CHCHD2	CLRN1	COL9A2	CRYM	CYP19A1	DHCR24
CCND2	CDKN2A	CHD2	CNGA3	COL9A3	CSF1R	CYP1B1	DHCR7
CCNH	CDKN2B-AS1	CHD4	CNGB1	COLEC11	CSF3R	CYP21A2	DHDDS
CCNO	CDON	CHD7	CNGB3	COLQ	CSNK1D	CYP24A1	DHFR
CCT7	CDSN	CHD8	CNNM2	COMP	CSNK2A1	CYP27A1	DHH
CD151	CDT1	CHEK2	CNNM4	COQ2	CSPP1	CYP4F22	DHODH

DHTKD1	DNMT3A	DYNC2H1	ELN-AS1	ERMARD	FANCA	FGFR3	FUCA1
DIABLO	DNMT3B	DYNC2LI1	ELOVL4	ESCO2	FANCC	FGFR4	FUS
DIAPH1	DOCK2	DYRK1A	ELOVL5	ESPN	FANCE	FH	FUT1
DICER1	DOCK6	DYRK1B	ELP1	ESR1	FANCF	FIBP	FUZ
DIPK1A	DOCK7	DYSF	ELP2	ESRRB	FANCG	FIG4	FXN
DIS3L2	DOCK8	EARS2	ELP4	ETFA	FANCI	FKBP10	FXYD6-FXYD2
DLD	DOLK	EBF3	EMG1	ETFB	FANCM	FKBP14	FZD4
DLL3	DONSON	ECE1	EML1	ETFDH	FAR1	FKRP	FZD6
DLL4	DPAGT1	ECEL1	EMP2	ETHE1	FARS2	FKTN	G6PC
DLX3	DPM1	ECHS1	ENG	ETV6	FAS	FLAD1	G6PC3
DLX5	DPM2	ECM1	ENPP1	EVC2	FASLG	FLCN	GAA
DMGDH	DPM3	EDARADD	ENTPD5	EXOSC3	FASTKD2	FLG	GABRA1
DMP1	DPRX	EDC3	EOGT	EXOSC8	FAT4	FLNB	GABRB1
DMXL2	DPY19L2	EDN1	EP300	EXT1	FBLN5	FLNC	GABRB3
DNA2	DPYD	EDN3	EPAS1	EXT2	FBN1	FLNC-AS1	GABRG2
DNAAF1	DPYS	EDNRB	EPB41	EYA1	FBN2	FLRT3	GAL
DNAAF2	DRAM2	EEF1A2	EPB41L4A	EYA4	FBP1	FLT3	GALC
DNAAF3	DRC1	EEF2	EPB42	EYS	FBXL3	FLT4	GALE
DNAAF4	DRD4	EFEMP2	EPCAM	EZH2	FBXL4	FLVCR1	GALK1
DNAAF5	DSC2	EFTUD2	EPG5	F10	FBXO31	FLVCR2	GALNS
DNAH1	DSC3	EGF	EPHB4	F11	FBXO38	FMO3	GALNT14
DNAH11	DSE	EGFR	EPHX2	F12	FBXO7	FN1	GALNT3
DNAH5	DSG1	EGFR-AS1	EPM2A	F13A1	FECH	FOLR1	GALNT4
DNAH8	DSG1-AS1	EGLN1	EPS8	F13B	FERMT1	FOXC1	GALT
DNAH8-AS1	DSG2	EGR2	EPX	F2	FERMT3	FOXC2	GAMT
DNAI1	DSG2-AS1	EHBP1	ERBB2	F5	FEZF1	FOXE1	GAN
DNAI2	DSG4	EHHADH	ERBB3	F7	FEZF1-AS1	FOXF1	GANAB
DNAJB2	DSP	EHMT1	ERBB4	FA2H	FGA	FOXG1	GARS1
DNAJB5	DSPP	EIF2AK1	ERCC1	FADD	FGB	FOXL2	GAS8
DNAJB6	DST	EIF2AK3	ERCC2	FAH	FGD4	FOXN1	GATA3
DNAJC19	DTNA	EIF2AK4	ERCC3	FAM111A	FGF10	FOXP2	GATA4
DNAJC21	DTNBP1	EIF2B1	ERCC4	FAM111B	FGF14	FOXRED1	GATA6
DNAJC5	DUOX2	EIF2B2	ERCC5	FAM120AOS	FGF17	FRAS1	GATAD1
DNAJC6	DUOXA2	EIF2B3	ERCC6	FAM126A	FGF20	FREM1	GATAD2B
DNAL1	DUPD1	EIF4A3	ERCC6L2	FAM161A	FGF23	FREM2	GATM
DNASE1	DUSP6	EIF4G1	ERCC8	FAM167A	FGF3	FRRS1L	GBA
DNM1	DVL1	ELANE	ERCC8-AS1	FAM20A	FGF8	FSHB	GBA2
DNM1L	DVL3	ELMO2	ERF	FAM20C	FGF9	FSHR	GBE1
DNM2	DYM	ELMOD3	ERLIN1	FAM83H	FGFR1	FTL	GCDH
DNMT1	DYNC1H1	ELN	ERLIN2	FAM98C	FGFR2	FTO	GCH1

GCK	GLRX5	GPT2	HBB	HOXA13	IER3IP1	IMPG2	IYD
GCLC	GLUD1	GPX4	HBD	HOXA2	IFIH1	INF2	JAG1
GCM2	GLUL	GREM2	HBG2	HOXB1	IFITM5	ING1	JAGN1
GCSH	GLYCTK	GRHL2	HCN1	HOXB13	IFNAR2	INPP5E	JAK1
GDAP1	GM2A	GRHL3	HCN4	HOXD13	IFNGR1	INPPL1	JAK3
GDF1	GMNN	GRHPR	HCRT	HPCA	IFNGR2	INS	JAM3
GDF2	GMPPB	GRIN1	HDC	HPD	IFT122	INS-IGF2	JMJD8
GDF3	GNA11	GRIN2A	HELLS	HPGD	IFT140	INSL3	JUP
GDF5	GNAI3	GRIN2B	HEPACAM	HPS1	IFT172	INSL6	KAAG1
GDNF	GNAL	GRIN2D	HERC1	HPS3	IFT27	INSR	KALRN
GFAP	GNAO1	GRIP1	HERC2	HPS4	IFT43	INVS	KANK2
GFER	GNAQ	GRM6	HES7	HPS5	IFT52	IQCB1	KANSL1
GFI1	GNAS	GRN	HEXA	HPS6	IFT74	IQCE	KARS1
GFI1B	GNAT1	GRXCR1	HEXB	HPSE2	IFT81	IRAK4	KAT6A
GFM2	GNAT2	GRXCR2	HFE	HR	IGF1	IRF1	KAT6B
GFPT1	GNB1	GSC	HFM1	HRAS	IGF2R	IRF4	KATNB1
GGCX	GNB3	GSDME	HGD	HSD11B1	IGHMBP2	IRF5	KCNA1
GH1	GNB4	GSS	HGF	HSD11B2	IGSF3	IRF6	KCNA2
GHR	GNE	GTF2E2	HGSNAT	HSD17B3	IHH	IRF7	KCNA5
GHRHR	GNMT	GTF2H5	HIBCH	HSD17B4	IKBKB	IRF8	KCNB1
GIGYF2	GNPAT	GTF2IRD1	HIKESHI	HSD3B2	IKZF1	IRGM	KCNC1
GIPC3	GNPTAB	GUCA1A	HINT1	HSD3B7	IL10RA	IRX5	KCNC3
GJA1	GNPTG	GUCY1A1	HIVEP2	HSPA1A	IL10RB	ISCA2	KCND3
GJA3	GNRH1	GUCY2C	HJV	HSPA1L	IL11RA	ISCU	KCNE1
GJA5	GNRHR	GUCY2D	HK1	HSPA9	IL12RB1	ISG15	KCNE2
GJA8	GNS	GUF1	HLCS	HSPB1	IL13	ITCH	KCNH1
GJB2	GORAB	GUSB	HMBS	HSPB8	IL17F	ITGA2B	KCNH2
GJB3	GOSR2	GYG1	HMGCL	HSPD1	IL17RA	ITGA3	KCNJ1
GJB4	GP1BA	GYS1	HMGCS2	HSPG2	IL17RC	ITGA7	KCNJ10
GJB6	GP1BB	GYS2	HNF1A	HTR1A	IL1B	ITGA8	KCNJ11
GJC2	GP9	H6PD	HNF1B	HTRA1	IL1RN	ITGB2	KCNJ2
GLB1	GPC6	HACE1	HNF4A	HTRA2	IL21R	ITGB3	KCNJ5
GLDC	GPC6-AS2	HADH	HNMT	HYAL1	IL2RA	ITGB4	KCNJ6
GLDN	GPD1	HADHB	HNRNPA1	HYDIN	IL36RN	ITGB6	KCNK3
GLI2	GPI	HAMP	HNRNPA2B1	IBA57	IL4R	ITK	KCNK9
GLI3	GPIHBP1	HARS1	HNRNPK	ICAM4	IL7R	ITPA	KCNMA1
GLIS2	GPNMB	HARS2	HOGA1	ICK	ILDR1	ITPKC	KCNMA1-AS1
GLMN	GPR179	HAVCR2	HOMER2	IDH2	IMPAD1	ITPR1	KCNN4
GLRA1	GPR68	HAX1	HOXA1	IDH3B	IMPDH1	ITSN1	KCNQ1
GLRB	GPSM2	HBA2	HOXA11	IDUA	IMPG1	IVD	KCNQ1-AS1

KCNQ1OT1	KLHL41	LARGE1	LOC101448202	LOC105375087	LOXL1	MAP2K1	MEFV
KCNQ2	KLHL7	LARS2	LOC101927188	LOC105375258	LPAR6	MAP2K2	MEGF10
KCNQ3	KLK4	LBR	LOC101927318	LOC105375554	LPIN1	MAP3K1	MEGF8
KCNQ4	KMT2A	LCA5	LOC101927513	LOC105375668	LPIN2	MAP3K7	MEN1
KCNT1	KMT2B	LCAT	LOC101927751	LOC105375669	LPL	MAPK1	MERTK
KCNV2	KMT2D	LCK	LOC101928008	LOC105376294	LRIG2	MAPK7	MESP2
KCTD1	KNL1	LCT	LOC101928462	LOC105378311	LRIT3	MAPKBP1	MEST
KCTD17	KPTN	LDB3	LOC101928477	LOC105378353	LRMDA	MAPRE2	MET
KCTD3	KRAS	LDLR	LOC101928861	LOC105378437	LRP2	MAPT	METTL23
KCTD7	KRIT1	LDLRAP1	LOC101929270	LOC105378525	LRP4	MARS1	MFAP5
KERA	KRT1	LDLR-AS1	LOC101930593	LOC105379077	LRP5	MARS2	MFN2
KHDC3L	KRT12	LEMD3	LOC102723409	LOC105379841	LRP6	MARVELD2	MFRP
KHK	KRT13	LEP	LOC102723439	LOC107984190	LRP8	MASP1	MFSD2A
KIAA0556	KRT14	LEPR	LOC102723566	LOC107984334	LRPAP1	MAT1A	MFSD8
KIAA0586	KRT16	LGI1	LOC102723692	LOC107984500	LRPPRC	MATN3	MGAT2
KIAA0753	KRT17	LHB	LOC102723869	LOC107984585	LRRC6	MATN4	MGME1
KIF11	KRT18	LHCGR	LOC102723945	LOC107984670	LRRCC1	MATR3	MGP
KIF14	KRT2	LHFPL5	LOC102723999	LOC107984763	LRRK2	MAX	MHRT
KIF1A	KRT25	LHX3	LOC102724262	LOC107985009	LRSAM1	MBD5	MIB1
KIF1B	KRT3	LIAS	LOC105369543	LOC107985023	LRTOMT	MBL2	MICU1
KIF1C	KRT4	LIFR	LOC105369668	LOC107985154	LTA	MBOAT7	MINPP1
KIF21A	KRT5	LIG4	LOC105369696	LOC107985290	LTBP2	MC2R	MIP
KIF22	KRT6A	LIMS2	LOC105369745	LOC107985291	LTBP3	MC4R	MIR6886
KIF2A	KRT6B	LINS1	LOC105369752	LOC107986432	LTBP4	MCCC1	MIR96
KIF5A	KRT6C	LIPA	LOC105369759	LOC107986469	LURAP1L-AS1	MCCC2	MITF
KIF5C	KRT71	LIPC	LOC105369798	LOC107986528	LYRM7	MCEE	MKKS
KIF7	KRT8	LIPH	LOC105369801	LOC107986608	LYZ	MCFD2	MKRN3
KIFBP	KRT83	LITAF	LOC105370042	LOC107986633	LZTFL1	MCIDAS	MKS1
KIRREL2	KRT9	LMBR1	LOC105370538	LOC107987102	LZTR1	MCM2	MLC1
KISS1	KYNU	LMBRD1	LOC105370627	LOC107987115	LZTS1	MCM8	MLH1
KISS1R	L2HGDH	LMF1	LOC105370752	LOC112267902	MAD1L1	MCM9	MLH3
KITLG	LACC1	LMNA	LOC105371049	LOC112267905	MAD2L2	MCOLN1	MLPH
KIZ	LAMA1	LMNB2	LOC105371251	LOC112267935	MAF	MCPH1	MLYCD
KLF1	LAMA2	LMOD3	LOC105371492	LOC112268118	MAFB	MDH2	MMAB
KLF11	LAMA3	LMX1B	LOC105371493	LOC112268182	MAG	MECOM	MMACHC
KLF6	LAMB1	LOC100130744	LOC105371567	LOC115308161	MAGEL2	MED13L	MMADHC
KLHDC8B	LAMB2	LOC100287944	LOC105371858	LOC647211	MAK	MED17	MME
KLHL10	LAMB3	LOC100506071	LOC105372146	LONP1	MALT1	MED23	MMP13
KLHL3	LAMC2	LOC100506321	LOC105372273	LOX	MAN1B1	MEF2A	MMP14
KLHL40	LAMC3	LOC100507346	LOC105372797	LOXHD1	MAN2B1	MEF2C	MMP2

MMP21	MVD	NAXE	NEXN	NR1H3	ORAI1	PCDH15	PEX7
MMUT	MVK	NBAS	NF1	NR1H4	ORC1	PCNA	PFKM
MNX1-AS2	MXI1	NBEAL2	NF2	NR2E3	ORC4	PCNT	PFN1
MOCOS	MYBPC1	NBN	NFIX	NR2F1	ORC6	PCSK1	PGAM2
MOCS1	MYBPC3	NCF1	NFKB2	NR2F2	OSBPL2	PCSK9	PGAP1
MOCS2	MYC	NCF2	NFKBIA	NR3C1	OSGEP	PCYT1A	PGAP2
MOG	MYCN	NCF4	NFU1	NR3C2	OSMR	PCYT2	PGAP3
MOGS	MYD88	NCLN	NHLRC1	NR5A1	OSTM1	PDCD10	PGBD3
MORC2	MYEF2	NCSTN	NHP2	NRAS	OTOA	PDE10A	PGM1
MPC1	MYH11	NDE1	NIN	NRL	OTOF	PDE11A	PGM3
MPDU1	MYH14	NDRG1	NIPA1	NRXN1	OTOG	PDE3A	PHGDH
MPDZ	MYH2	NDST1	NIPAL4	NSD1	OTOGL	PDE4D	PHKB
MPI	MYH3	NDUFA13	NIPBL	NSMCE3	OTOP2	PDE6A	PHKG2
MPL	MYH6	NDUFA2	NKX2-1	NSUN2	OTULIN	PDE6B	PHOX2A
MPLKIP	MYH7	NDUFA6	NKX2-5	NSUN6	OTX2	PDE6C	PHYH
MPO	MYH9	NDUFAF1	NKX2-6	NT5C2	OVOL2	PDE6D	PHYKPL
MPV17	MYL2	NDUFAF2	NLRC4	NT5C3A	OXCT1	PDE6H	PI4KA
MPZ	MYL3	NDUFAF4	NLRP1	NTF4	P2RX2	PDE8B	PIBF1
MPZL2	MYL4	NDUFAF5	NLRP3	NTHL1	P3H1	PDGFRB	PIEZO1
MRAP	MYLK	NDUFAF6	NLRP7	NTRK1	P4HB	PDHB	PIEZO2
MRE11	MYMK	NDUFS1	NMNAT1	NTRK2	PACRG	PDHX	PIGG
MRI1	MYNN	NDUFS2	NNT	NUBPL	PAFAH1B1	PDP1	PIGN
MS4A2	MYO15A	NDUFS3	NOBOX	NUDT2	PAH	PDSS1	PIGO
MSH2	MYO18B	NDUFS4	NOC3L	NUP107	PALB2	PDSS2	PIGT
MSH3	MYO1E	NDUFS6	NOD2	NUP155	PAM16	PDX1	PIGV
MSH6	MYO3A	NDUFS7	NODAL	NUP205	PANK2	PDYN	PIGW
MSR1	MYO5B	NDUFS8	NOG	NUP62	PAPSS2	PEPD	PIH1D2
MSRB3	MYO6	NDUFV1	NOP10	NUP93	PARK7	PER3	PIK3CA
MSX2	MYO7A	NDUFV2	NOTCH1	NUP98	PARN	PET100	PIK3CD
MTFMT	MYOC	NEB	NOTCH2	OAT	PAX2	PEX1	PIK3R1
MTHFR	MYOM1	NECTIN1	NOTCH3	OBSL1	PAX3	PEX10	PIK3R2
MTMR10	MYOT	NECTIN4	NPC1	OCA2	PAX4	PEX11B	PINK1
MTMR2	MYOZ2	NEDD4L	NPC2	OCLN	PAX6	PEX12	PIP5K1C
MTOR	MYPN	NEFH	NPHP1	ODAPH	PAX8	PEX13	PITX1
MTPAP	MYT1L	NEFL	NPHP4	OPA1	PAX9	PEX14	PITX2
MTR	NADK2	NEK2	NPHS1	OPA3	PC	PEX16	PKD1
MTRR	NAGA	NEK8	NPPA	OPCML	PCARE	PEX2	PKD1L1
MTTP	NAGLU	NEK9	NPR2	OPLAH	PCBD1	PEX26	PKD2
MUSK	NAGS	NEU1	NPRL2	OPN1SW	PCCA	PEX5	PKHD1
MUTYH	NALCN	NEUROG3	NPRL3	OPTN	PCDH12	PEX6	PKLR

PKP1	POLK	PRKCSH	PTPRQ	RBM20	RNASEH1	RSPO4	SCN4B
PKP2	POLR1A	PRKDC	PTRH2	RBM28	RNASEH2A	RSPRY1	SCN5A
PLA2G6	POLR1C	PRKG1	PTS	RBM8A	RNASEH2B	RTEL1	SCN8A
PLA2G7	POLR1D	PRKN	PUF60	RBP4	RNASEH2C	RTN2	SCN9A
PLCB4	POLR2F	PRLR	PURA	RBPJ	RNASEL	RTN4IP1	SCNN1A
PLCE1	POLR3A	PRNP	PUS1	RCBTB1	RNASET2	RTN4R	SCNN1B
PLCE1-AS1	POLR3B	PROC	PUS3	RD3	RNF125	RTTN	SCNN1G
PLCG1-AS1	POMC	PRODH	PXDN	RDH11	RNF168	RUBCN	SCO1
PLCG2	POMGNT1	PROKR2	PYCR1	RDH12	RNF170	RUNX1	SCP2
PLEC	POMGNT2	PROM1	PYCR2	RDH5	RNF213-AS1	RUNX2	SCYL1
PLEKHG5	POMK	PROP1	PYGL	RDX	RNF216	RXYLT1	SDCCAG8
PLEKHM1	POMT1	PROS1	PYGM	RECQL	RNF6	RXYLT1-AS1	SDHA
PLG	POMT2	PRPF3	PYROXD1	RECQL4	RNU4ATAC	RYR1	SDHAF2
PLK4	PON1	PRPF31	QDPR	REEP1	ROBO3	RYR2	SDHB
PLN	POR	PRPF4	RAB18	REEP2	ROBO4	S1PR2	SDHC
PLOD1	POU4F3	PRPF6	RAB23	REEP6	ROGDI	SACS	SDHD
PLOD2	PPARG	PRPF8	RAB27A	RELA	ROR2	SAG	SEC23A
PLOD3	PPIB	PRPH2	RAB28	RELN	RORB	SALL1	SEC23B
PLPBP	PPOX	PRRT2	RAB33B	RELT	RORC	SALL2	SEC24D
PMFBP1	PPP1CB	PRRX1	RAB3GAP1	REN	RP1	SALL4	SEC61A1
PMM2	PPP1R15B	PRSS1	RAB3GAP2	RERE	RP1L1	SAMD9	SEC63
PMP22	PPP2R1A	PRSS12	RAB7A	RET	RPE65	SAMD9L	SECISBP2
PMPCA	PPP2R1B	PRSS56	RAC2	RETREG1	RPGRIP1	SAMHD1	SELENON
PMS2	PPP2R5D	PRUNE1	RAD21	RFT1	RPGRIP1L	SAR1B	SEMA4A
PMVK	PPT1	PRX	RAD50	RFX5	RPIA	SARDH	SEPTIN12
PNKP	PRCD	PSAP	RAD51	RFX6	RPL21P4	SARS2	SEPTIN9
PNP	PRDM12	PSAT1	RAD51C	RFXANK	RPL26	SASH1	SERAC1
PNPLA1	PRDM16	PSEN1	RAD51D	RFXAP	RPS10	SATB2	SERPINA1
PNPLA2	PRDM5	PSEN2	RAG1	RGS9	RPS17	SBDS	SERPINA6
PNPLA6	PRDM6	PSMB8	RAG2	RHAG	RPS19	SBF1	SERPINB6
PNPLA8	PRF1	PSPH	RAI1	RHBDF2	RPS24	SBF2	SERPINB7
PNPO	PRICKLE1	PSTPIP1	RAPSN	RHO	RPS26	SC5D	SERPINB8
PNPT1	PRKACA	PTCH1	RARB	RIN2	RPS29	SCARF2	SERPINC1
POC1B	PRKACG	PTCH2	RARS1	RIPK4	RPS7	SCN11A	SERPIND1
POFUT1	PRKAG2	PTDSS1	RARS2	RIPOR2	RPSA	SCN1A	SERPINF1
POGZ	PRKAG3	PTEN	RASA1	RIT1	RRM2B	SCN1B	SERPINF2
POLD1	PRKAR1A	PTGIS	RAX	RLBP1	RSPH1	SCN2B	SERPING1
POLE	PRKCD	PTH	RB1	RMND1	RSPH3	SCN3A	SERPINH1
POLG	PRKCG	PTHLH	RBBP8	RMRP	RSPH4A	SCN3B	SERPINI1
POLG2	PRKCH	PTPN11	RBCK1	RNASE4	RSPH9	SCN4A	SETBP1

SETD1A	SLC12A5	SLC34A1	SMAD4	SPAG1	STAR	TAC3	TEAD1
SETD2	SLC12A6	SLC34A3	SMAD9	SPAG8	STAT1	TACR3	TECPR2
SETD5	SLC13A5	SLC35A3	SMARCA2	SPARC	STAT2	TACSTD2	TECR
SETX	SLC17A5	SLC35C1	SMARCA4	SPART	STAT3	TAF2	TECTA
SEZ6	SLC17A9	SLC35D1	SMARCAL1	SPAST	STAT5B	TAF6	TEK
SF3B4	SLC18A3	SLC37A4	SMARCB1	SPATA6L	STEAP3	TALDO1	TENM3
SFRP4	SLC19A1	SLC38A8	SMARCD2	SPATA7	STIL	TANGO2	TENM4
SFTPA2	SLC1A2	SLC39A14	SMARCE1	SPDL1	STIM1	TARDBP	TERC
SFTPB	SLC1A3	SLC39A4	SMC3	SPECC1L	STING1	TARID	TERT
SFTPC	SLC20A2	SLC39A5	SMCHD1	SPEG	STK11	TAS2R16	TEX15
SGCA	SLC22A12	SLC3A1	SMG9	SPG11	STK4	TAT	TF
SGCB	SLC22A18	SLC40A1	SMN1	SPG21	STOX1	TBATA	TFAM
SGCD	SLC22A5	SLC45A2	SMN2	SPG7	STRA6	TBC1D20	TFAP2A
SGCE	SLC24A4	SLC46A1	SMO	SPINK1	STRC	TBC1D24	TFAP2A-AS2
SGCG	SLC25A13	SLC4A1	SMOC1	SPINK5	STT3A	TBC1D7	TFAP2B
SGSH	SLC25A15	SLC4A11	SMOC2	SPINT2	STT3B	TBCD	TFG
SH2B3	SLC25A19	SLC52A2	SMPD1	SPR	STUB1	TBCE	TFR2
SH3PXD2B	SLC25A20	SLC52A3	SNAP25	SPRED1	STX11	TBK1	TG
SH3TC2	SLC25A22	SLC5A1	SNAP29	SPRTN	STX1B	TBL1XR1	TGDS
SHANK2	SLC25A3	SLC5A2	SNCA	SPRY4	STX7	TBX1	TGFB1
SHANK3	SLC25A32	SLC5A5	SNCB	SPTA1	STXBP1	TBX18	TGFB2
SHH	SLC25A38	SLC5A7	SNORD118	SPTAN1	STXBP2	TBX19	TGFB3
SHOC2	SLC25A4	SLC6A17	SNRNP200	SPTB	SUCLA2	TBX20	TGFBI
SHPK	SLC25A42	SLC6A19	SNRPB	SPTBN2	SUCLG1	TBX3	TGFBR
SI	SLC25A46	SLC6A3	SNRPE	SPTLC1	SUFU	TBX4	TGFBR1
SIAE	SLC26A2	SLC6A5	SNTA1	SPTLC2	SUGCT	TBX5	TGIF1
SIGMAR1	SLC26A3	SLC6A9	SNX10	SPTLC3	SUMF1	TBX6	TGM1
SIK1	SLC26A4	SLC7A14	SNX14	SQSTM1	SUN5	TBXT	TGM5
SIL1	SLC26A5	SLC7A7	SOBP	SRC	SUOX	TCAP	TGM6
SIN3A	SLC26A8	SLC7A9	SOD1	SRCAP	SURF1	TCF12	TH
SIX1	SLC27A4	SLC9A9	SOHLH1	SRD5A2	SYCE1	TCF3	TH2LCRR
SIX3	SLC28A1	SLCO1B1	SON	SRD5A3	SYCE2	TCF4	THAP1
SIX5	SLC29A3	SLCO2A1	SOS1	SRGAP1	SYCP3	TCIRG1	THBD
SIX6	SLC2A1	SLFN14	SOS2	SRI	SYNE1	TCN2	THCAT158
SKI	SLC2A10	SLITRK1	SOST	ST14	SYNE1-AS1	TCOF1	THOC6
SLC10A2	SLC2A2	SLITRK6	SOX10	ST3GAL3	SYNE4	TCTN1	THRA
SLC11A2	SLC2A9	SLMO2-ATP5E	SOX11	ST3GAL5	SYNGAP1	TCTN2	THSD1
SLC12A1	SLC30A10	SLURP1	SOX18	STAC3	SYNJ1	TCTN3	TIA1
SLC12A3	SLC30A2	SLX4	SOX5	STAMBP	SZT2	TDP1	TIMM50
SLC12A4	SLC33A1	SMAD3	SOX9	STAP1	TAB2	TDP2	TIMP3

TINF2	TNIK	TRIM44	TUBA1A	UROC1	WDPCP	YARS1	
TJP2	TNNC1	TRIO	TUBA3E	UROD	WDR19	YARS2	
TK2	TNNI2	TRIOBP	TUBA4A	UROS	WDR34	YME1L1	
TLE6	TNNI3	TRIP11	TUBA8	USB1	WDR35	YY1AP1	
TLL1	TNNT1	TRIP4	TUBB	USH1C	WDR36	ZAP70	
TLR2	TNNT2	TRMU	TUBB1	USH1G	WDR45B	ZBTB16	
TLR5	TNNT3	TRPA1	TUBB2A	USH2A	WDR60	ZBTB18	
TMC1	TNPO3	TRPC3	TUBB2B	USP53	WDR62	ZBTB24	
TMCO1	TNXB	TRPC6	TUBB3	USP8	WDR66	ZBTB42	
TMEM107	TOP3A	TRPM1	TUBB4A	UTP14C	WDR72	ZC3H14	
TMEM126A	TOPORS	TRPM4	TUBB8	VAC14	WDR73	ZDHHC24	
TMEM126B	TOR1AIP1	TRPM6	TUBG1	VAC14-AS1	WDR81	ZEB1	
TMEM127	TP53	TRPM7	TUBGCP4	VANGL1	WDR93	ZEB2	
TMEM138	TP53BP1	TRPS1	TUBGCP6	VAPB	WEE2	ZFP57	
TMEM165	TP63	TRPV3	TULP1	VARS2	WFS1	ZFPM2-AS1	
TMEM199	TPCN2	TRPV4	TUSC3	VAX1	WHRN	ZFYVE26	
TMEM216	TPH2	TSC1	TWIST1	VCAN-AS1	WNK1	ZIC1	
TMEM231	TPI1	TSC2	TWIST2	VCL	WNK4	ZIC2	
TMEM237	TPK1	TSEN15	TWNK	VCP	WNT1	ZMPSTE24	
TMEM240	TPM1	TSEN2	TXNDC15	VEGFA	WNT10A	ZMYND10	
TMEM256	TPM2	TSEN34	TXNL4A	VEGFC	WNT10B	ZMYND11	
TMEM43	TPM3	TSEN54	TYK2	VHL	WNT3	ZMYND15	
TMEM67	TPO	TSHB	TYMP	VIPAS39	WNT4	ZNF335	
TMEM70	TPP1	TSHR	TYR	VPS11	WNT5A	ZNF408	
TMEM92	TPRN	TSHZ1	TYROBP	VPS13A	WNT7A	ZNF469	
TMEM98	TPTE2P5	TSPAN12	TYRP1	VPS13B	WRAP53	ZNF627	
TMEM99	TRAC	TSPYL1	UBE3A	VPS13C	WRN	ZNF644	
TMIE	TRAF3IP1	TTBK1	UBE3B	VPS33A	WT1	ZNF687	
TMPRSS15	TRAF3IP2	TTBK2	UBIAD1	VPS33B	WWOX	ZNHIT3	
TMPRSS3	TRAIP	TTC19	UBN1	VPS35	XDH	ZP1	
TMPRSS6	TRAPPC11	TTC21B	UBR1	VPS45	XIRP1	ZSWIM6	
TMTC3	TRAPPC3	TTC25	UCP3	VPS53	XPA		
TNC	TRAPPC9	TTC37	UMOD	VRK1	XPC		
TNF	TRDN	TTC7A	UNC13D	VSX1	XPNPEP3		
TNFAIP3	TREM2	TTC8	UNC45B	VSX2	XRCC2		
TNFRSF11A	TRHR	TTI2	UNG	VWA3B	XRCC3		
TNFRSF11B	TRIM14	TTLL5	UPB1	VWF	XRCC4		
TNFRSF13B	TRIM2	TTN	UQCC3	WAC	XYLT1		
TNFRSF1A	TRIM32	TTPA	UQCRQ	WASHC4	XYLT2		
TNFSF11	TRIM37	TTR	URAHP	WASHC5	YAP1		
