# Supplementary material for: Genetic profiles of 103,106 individuals in the Taiwan Biobank provide insights into the health and history of Han Chinese
Source: NPJ Genom Med. 2021 Feb 11;6:10. doi: 10.1038/s41525-021-00178-9 (PMC7878858; doi:10.1038/s41525-021-00178-9)
Supplement: Supplementary file 2 — Reporting Summary [file 41525_2021_178_MOESM2_ESM.pdf]

nature research

Corresponding author(s): Pui-Yan Kwok

Last updated by author(s): Dec 20, 2020

Reporting Summary

Nature Research wishes to improve the reproducibility of the work that we publish. This form provides structure for consistency and transparency in reporting. For further information on Nature Research policies, see our Editorial Policies and the Editorial Policy Checklist.

Statistics

For all statistical analyses, confirm that the following items are present in the figure legend, table legend, main text, or Methods section.

n/a	Confirmed	
[ ]	[x]	The exact sample size (n) for each experimental group/condition, given as a discrete number and unit of measurement
[x]	[ ]	A statement on whether measurements were taken from distinct samples or whether the same sample was measured repeatedly
[ ]	[x]	The statistical test(s) used AND whether they are one- or two-sided<br>*Only common tests should be described solely by name; describe more complex techniques in the Methods section.*
[x]	[ ]	A description of all covariates tested
[x]	[ ]	A description of any assumptions or corrections, such as tests of normality and adjustment for multiple comparisons
[ ]	[x]	A full description of the statistical parameters including central tendency (e.g. means) or other basic estimates (e.g. regression coefficient) AND variation (e.g. standard deviation) or associated estimates of uncertainty (e.g. confidence intervals)
[ ]	[x]	For null hypothesis testing, the test statistic (e.g. F, t, r) with confidence intervals, effect sizes, degrees of freedom and P value noted<br>*Give P values as exact values whenever suitable.*
[x]	[ ]	For Bayesian analysis, information on the choice of priors and Markov chain Monte Carlo settings
[x]	[ ]	For hierarchical and complex designs, identification of the appropriate level for tests and full reporting of outcomes
[x]	[ ]	Estimates of effect sizes (e.g. Cohen's d, Pearson's r), indicating how they were calculated

Our web collection on statistics for biologists contains articles on many of the points above.

Software and code

Policy information about availability of computer code

Data collection	N/A
Data analysis	Open Source tools: GATK v3.8-1; SHAPEIT2; IMPUTE2; Hibag R<br>No custom tools

For manuscripts utilizing custom algorithms or software that are central to the research but not yet described in published literature, software must be made available to editors and reviewers. We strongly encourage code deposition in a community repository (e.g. GitHub). See the Nature Research guidelines for submitting code & software for further information.

Data

Policy information about availability of data

All manuscripts must include a data availability statement. This statement should provide the following information, where applicable:

- Accession codes, unique identifiers, or web links for publicly available datasets
- A list of figures that have associated raw data
- A description of any restrictions on data availability

All data are available upon request from the databases used (as we have done for this study). As these are public databases, we are not authorized to repost them in aggregate. All the data we generated for this study are found in the supplementary materials.

Field-specific reporting

Please select the one below that is the best fit for your research. If you are not sure, read the appropriate sections before making your selection.

☒ Life sciences ☐ Behavioural & social sciences ☐ Ecological, evolutionary & environmental sciences

For a reference copy of the document with all sections, see nature.com/documents/nr-reporting-summary-flat.pdf

Life sciences study design

All studies must disclose on these points even when the disclosure is negative.

Sample size	Total sample number with whole genome sequencing = 1,492; total sample number with genotyping data = 103,106. This is not a statistical study to prove a certain hypothesis. It is rather a survey of samples from Taiwan to establish allele frequencies of known risk variants for various conditions in Taiwan. As such, the sample size was based on how many samples with relevant data are available from the public domain.
Data exclusions	None.
Replication	This is not a statistical study so replication is not applicable.
Randomization	Because this is not a statistical study, randomization is not appropriate.
Blinding	As a survey study of fully de-identified data, blinding is not necessary.

Reporting for specific materials, systems and methods

We require information from authors about some types of materials, experimental systems and methods used in many studies. Here, indicate whether each material, system or method listed is relevant to your study. If you are not sure if a list item applies to your research, read the appropriate section before selecting a response.

Materials & experimental systems

n/a	Involved in the study	
☒	☐	Antibodies
☒	☐	Eukaryotic cell lines
☒	☐	Palaeontology and archaeology
☒	☐	Animals and other organisms
☐	☒	Human research participants
☒	☐	Clinical data
☒	☐	Dual use research of concern

Methods

n/a	Involved in the study	
☒	☐	ChIP-seq
☒	☐	Flow cytometry
☒	☐	MRI-based neuroimaging

Human research participants

Policy information about studies involving human research participants

Population characteristics	Our dataset includes Taiwan Biobank participants (mostly Han Chinese) that are part of a public database.
Recruitment	The 103,106 Taiwanese subjects in this study were recruited by the Taiwan Biobank.
Ethics oversight	The Internal Review Board of the Taiwan Biobank.

Note that full information on the approval of the study protocol must also be provided in the manuscript.
